# Enhancing CO_2_ to Alcohol Conversion: Powerful Photocatalysts Based on TiO_2_–Cu(I)-Iodine-Pyridine One-Dimensional Coordination Polymers

**DOI:** 10.1021/acs.inorgchem.5c04083

**Published:** 2025-11-11

**Authors:** Julian Avila-Duran, Jon Napal, Fernando Aguilar-Galindo, Oscar Castillo, Pilar Amo-Ochoa

**Affiliations:** Δ Inorganic Chemistry Department, Faculty of Sciences, Autonomous University of Madrid (UAM), Madrid 28049, Spain; α Department of Organic and Inorganic Chemistry, University of the Basque Country (UPV/EHU), P.O. 644, Bilbao E-48080, Spain; β Chemistry Department, Faculty of sciences, Autonomous University of Madrid (UAM), Madrid 28049, Spain; Ω Institute for Advanced Research in Chemical Sciences (IAdChem), Autonomous University of Madrid (UAM), Madrid 28049, Spain

## Abstract

Coordination polymers (CPs) are promising materials for environmental applications, particularly in catalysis, due to their flexible structures, tunable electronic properties, and adaptable surface chemistry. This study reports the one-step, room-temperature synthesis of five 1D Cu­(I)-iodide-pyridine based CPs with the general formula [CuI­(L)]_*n*_, where L represents different pyridine derivatives: pyridine (**CP1**), 3-methylpyridine (**CP2**), 4-methylpyridine (**CP3**), 2-amino-4-methylpyridine (**CP4**), and 2-chloro-4-methylpyridine (**CP5**). All of the compounds exhibit band gap energies around 3 eV, making them suitable candidates for photocatalytic applications. Indeed, the study investigates the photoreduction of CO_2_ to alcohols using a heterogeneous photocatalytic system consisting of TiO_2_ and varying proportions of CPs. The reactor design enables the rapid removal of produced alcohols, preventing them from being oxidized by TiO_2_ as sacrificial materials and thus achieving near-zero net alcohol production. The optimal **TiO** **_2_** **@CP** mixture, **TiO** **_2_** **@5%CP4**, demonstrated higher chemical stability due to the amine substituent on the pyridine, which facilitates hydrogen bonding between CP chains, and an enhanced ability to interact with CO_2_, as confirmed by adsorption experiments and DFT calculations. The optimized mixture achieved selective methanol production of 894 μg·g cat ^–1^·h ^–1^, significantly surpassing the benchmark photocatalytic system **TiO** **_2_** **@3%CuO** (318 μg·g cat ^–1^·h ^–1^). Furthermore, **TiO** **_2_** **@5%CP4** maintained stable photocatalytic performance over 10 h without noticeable degradation.

## Graphical abstract

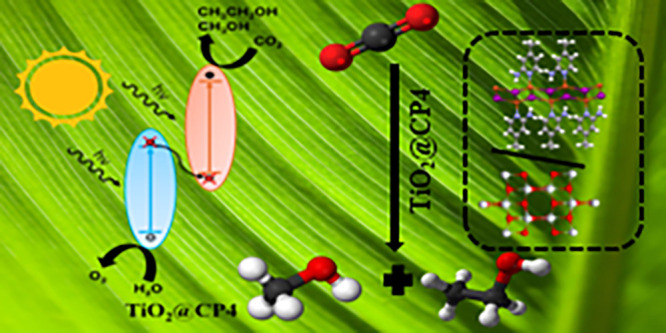

## 1 Introduction

Carbon dioxide (CO_2_) is the primary contributor to global climate change, accounting for over 64% of greenhouse gas emissions into the atmosphere.^1^ Excessive CO_2_ release has severely disrupted the natural carbon cycle, leading to major environmental consequences, most notably the intensification of the greenhouse effect.^2^ Consequently, CO_2_ capture and transformation technologies are being developed at a rapid pace to mitigate these issues. However, CO_2_ exhibits remarkable chemical stability primarily due to the strength of its C=O double bonds. As a result, any attempt to transform CO_2_ requires considerable energy input to overcome this thermodynamic barrier.^3^ Since carbon in CO_2_ is in its highest oxidation state, reducing it can yield a wide range of valuable products with lower oxidation states, including carbon monoxide, methane, and oxygenated liquid compounds such as methanol, ethanol, and formic acid.^4^ Various strategies have been developed for CO_2_ conversion, including chemical, enzymatic, thermochemical, electrochemical, biochemical, and photochemical methods.^5^ Among these, photocatalytic CO_2_ reduction is regarded as a sustainable and cost-effective approach, enabling the transformation of CO_2_ and H_2_O into hydrocarbon fuels using solar energy as the sole energy input.^6,7^ The first reports on photocatalytic CO_2_ reduction emerged almost simultaneously: Halmann demonstrated the formation of formic acid using a p-type gallium phosphide photoelectrode after 18 h of reaction,^8^ and Inoue et al. reported the production of methanol and formaldehyde using semiconductors such as TiO_2_, ZnO, CdS, GaP, and SiC in aqueous suspensions.^9^ After that, TiO_2_ has been extensively studied in CO_2_ photoreduction, due to its favorable optoelectronic characteristics, high thermal stability, low cost, and strong photocatalytic activity.^10,11^ Among its various crystalline forms, the anatase phase exhibits superior photoconductivity, with a bandgap of around 3.2 eV.^12^ Despite its advantages, TiO_2_ has certain limitations, primarily due to its relatively low quantum efficiency. This is largely due to a high recombination rate of up to 90% of the photogenerated electron–hole pairs during the photocatalytic process.^13^

To enhance the quantum efficiency of TiO_2_ and suppress charge recombination, various modification strategies have been explored, such as doping and forming heterojunctions with different cocatalysts.^14^ These approaches promote more efficient charge separation by facilitating the transfer of photogenerated electrons to the cocatalyst, thereby reducing the likelihood of electron–hole recombination. The cocatalyst also increases affinity toward the CO_2_ molecule and largely determines the selectivity of the reaction toward different products (CO, HCOOH, CH_4_, CH_3_OH, CH_3_CH_2_OH, etc.).^15–17^ A wide array of cocatalysts has been investigated for this purpose, including different metal sulfides,^18^ metal oxides,^19–24^ graphitic carbon nitride (g-C_3_N_4_),^25^ and metal halides.^26^ Among these, copper, CuI, and copper oxide cocatalysts have garnered particular attention due to their notable efficiency in facilitating alcohol production during CO_2_ photoreduction.^27–30^ However, the production efficiency and selectivity remain low by industry standards, prompting extensive research to identify the most appropriate TiO_2_ cocatalyst.

The application of coordination compounds (CCs) to CO_2_ photoreduction is emerging as their flexible and dynamic structures, tunable electronic properties, and adaptable surface chemistry are yielding highly promising results.^31^ Studies have been primarily focused on porous coordination polymers (PCPs), such as metal–organic frameworks (MOFs),^30,32,33^ as the source of e^–^/h^+^ pairs upon light irradiation.^34,35^ However, the most efficient MOFs for CO_2_ reduction rely on their doping or combination with nanoparticles of an inorganic cocatalyst (RuO_*x*_, Cu, Au, etc.).^36,37^ These inorganic cocatalysts are usually based on soft metals that can interact better with CO_2_ molecules and facilitate electron transfer to them more readily. Water-stable MOFs are usually based on a combination of secondary building units (SBUs) involving hard transition metal ions and carboxylate ligands. Therefore, they require a second cocatalyst or on a postsynthetic modification of the MOF to introduce a CO_2_ interaction site that interacts better with CO_2_, often by doping with softer metal atoms, ions, or complexes.^38,39^ The use of nonporous coordination polymers (NPCPs) has been less explored; however, these materials offer significant advantages, including greater structural diversity. Many NPCPs are also based on soft metal ions, which, when combined with appropriately chosen ligands, can enhance their ability to interact with CO_2_ compared with purely inorganic systems. This enables the development of efficient photocatalytic systems that couple well-established inorganic electron–hole (e^–^/h^+^) pair generators with CPs containing soft metal centers. Nonetheless, this strategy remains relatively underexplored and often relies on expensive or toxic metals.^40–44^ Therefore, it is desirable to develop photocatalysts based on more abundant and environmentally benign metals. Another crucial factor to consider for potential industrial applications is the simplicity of fabrication. However, few investigations focus on facile room-temperature synthesis.^45^

Considering the previous comments, this study explores the synergy that can be achieved by combining copper iodide (CuI) with organic ligands derived from pyridines. The Cu­(I), with a d^10^ configuration, tends to adopt a tetrahedral coordination, which is a stable motif compared to higher coordination numbers. In solid CuI, the low solubility and high melting point support the robustness of the Cu–I framework. Furthermore, when suitably strong ligands (e.g., N-donors) are coordinated, the Cu­(I) center is protected against oxidation and disproportionation, enabling stable complexes with bridging iodide frameworks (e.g., μ_3_–I or μ_4_–I bridges) that act to rigidify the lattice and suppress degradative pathways. This approach allows the sustainable, scalable one-step, room-temperature synthesis of air-stable 1D-NPCPs.^46^ These Cu­(I)-I-based CPs are insoluble in water, exhibit flexible and dynamic bonding and semiconductor properties, and can absorb visible light. Additionally, the Cu–I bonds are quite stable in water over long periods, especially when further stabilized by the presence of protecting methylpyridines.^47^ They also have well-established electrical and catalytic properties.^48–51^ The presence of aromatic rings and various functional groups imparts distinct polarities and enables diverse supramolecular interactions, which are expected to enhance the CO_2_ adsorption and interaction.

Specifically, five 1D CPs were designed and selected for this study. Each incorporates a Cu­(I)-I ladder chain motif, along with a pyridine derivative coordinated to the metal center (Scheme 1). Methyl groups were introduced at different positions to study their influence on the band gap, observing that the position of the substituent significantly modifies this parameter. The system with the lowest band gap was taken as a starting point for incorporating functional groups capable of establishing molecular interactions, to analyze how these interactions affect electronic properties and catalytic activity. These CPs exhibit semiconductor behavior and will be used as cocatalysts alongside TiO_2_ reference catalyst. The novelty of this work lies in the use of nonporous Cu­(I)-I coordination polymers to overcome the intrinsic limitations of TiO_2_, allowing for more efficient charge separation, greater CO_2_ activation, and the selective production of alcohols in aqueous media without sacrificial agents.

**Scheme 1:**
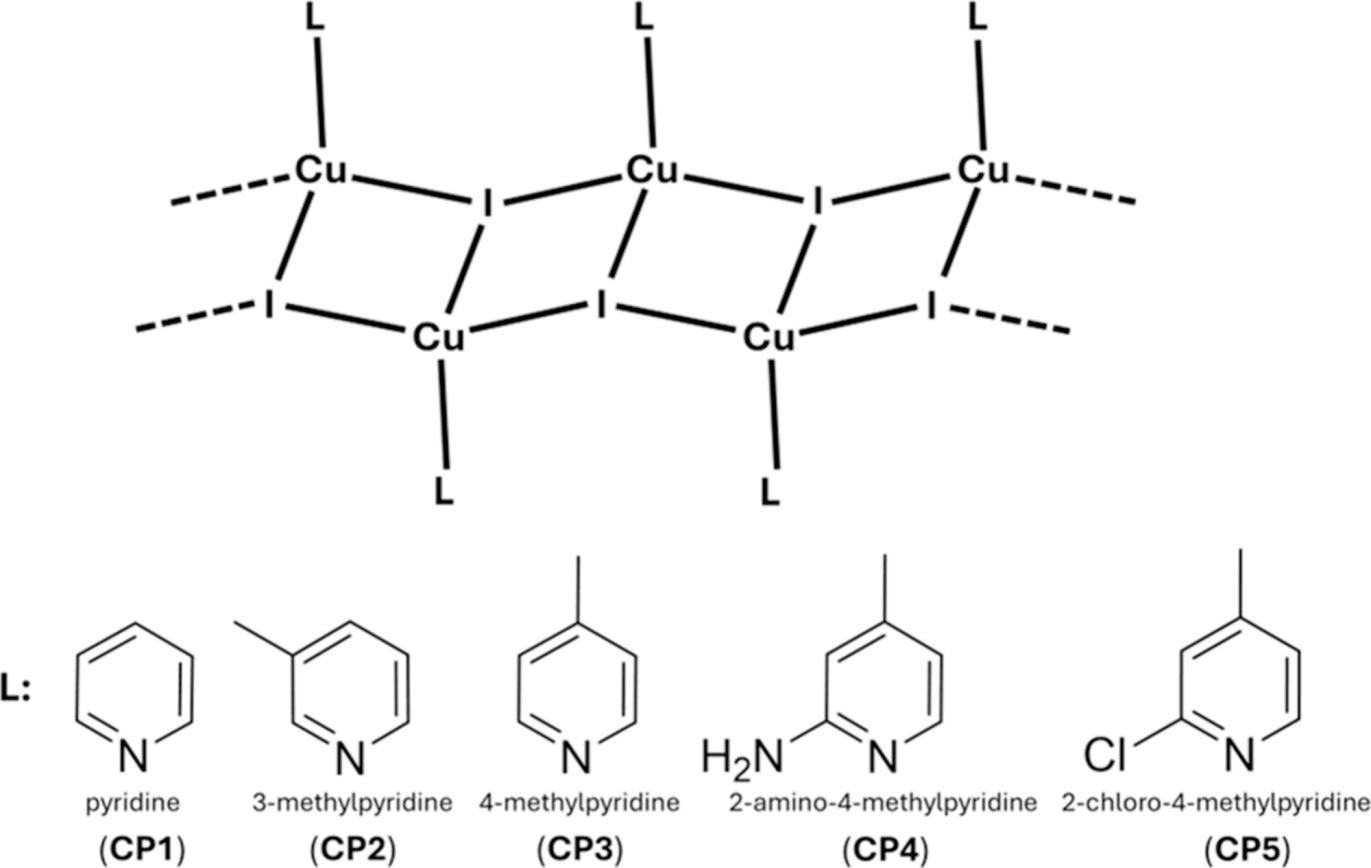
Cu­(I)-I Ladder Chain Motif along with a Pyridine Derivative Coordinated to the Metal Center

## 2 Experimental Section

### 2.1 Materials

All reagents were used as obtained from the suppliers. Ethanol (≥99.9%) was purchased from Scharlau. Pyridine (≥99.8%) was supplied by Prolabo. The supplier for 3-methylpyridine (≥99%), 2-amino-4-methylpyridine (≥99%), 2-chloro-4-methylpyridine (≥98%), copper­(I) iodide (≥99%), titanium­(IV) oxide, anatase (<25 nm, ≥99.7%), and potassium bicarbonate (≥99.5%) is Sigma-Aldrich. Potassium iodide (≥99.5%) was purchased from Fluka. 4-Methylpyridine (≥99%) was supplied by Thermo Scientific.

### 2.2 Synthesis

#### 2.2.1 [CuI­(L)]_*n*_ polycrystals (L= pyridine (**CP1**),^52^ 3-methylpyridine (**CP2**),^53^ 4-methylpyridine (**CP3**),^53^ 2-amino-4-methylpyridine (**CP4**), and 2-chloro-4-methylpyridine (**CP5**)

Solutions of CuI (190.5 mg, 1 mmol) in concentrated aqueous KI (4 g, 4 mL) are prepared under magnetic stirring (800 rpm) at room temperature. After that, and according to the compound being prepared, ligand of pyridine (79,1 mg, 1 mmol), 3-methylpyridine (93.1 mg, 1 mmol), 4-methylpyridine (93.1 mg, 1 mmol), 2-amino-4-methylpyridine (108 mg, 1 mmol), or 2-chloro-4-methylpyridine (128 mg, 1 mmol) are added. Immediately, white precipitates are formed on all syntheses, and the reactions are stirred at 25 °C for 15 min. The obtained precipitates are filtered off under vacuum and washed with concentrated aqueous KI (10 mL × 2) and H_2_O (15 mL × 3). Finally, the precipitates are dried under vacuum for 24 h. Yields: (**CP1**) 175 mg, yield: 65%, (**CP2**) 196 mg, yield: 69%; (**CP3**) 213 mg, yield: 75% (**CP4**) 203 mg, yield: 68%, and (**CP5**) 127 mg, yield: 40%, based on the monomer of each CP.

(**CP1**) Anal. Calcd for [CuI­(C_5_H_5_N)]_n_: C, 22.28; H, 1.87; N, 5.20; found: C, 22.12; H, 2.08; N, 5.23. IR (cm^–1^): 1597 (w), 1481 (w), 1442 (m), 1212 (w), 1146 (w), 1068 (w), 1038 (w), 1007 (w), 748 (m), 694 (s), and 632 (w).

(**CP2**) Anal. Calcd for [CuI­(C_6_H_7_N)]_n_: C, 25.41; H, 2.49; N, 4.94; found: C, 25.30; H, 2.60; N, 4.95. IR (cm^–1^): 1601 (w), 1478 (w), 1451 (w), 1416 (w), 1369 (w), 1188 (w), 1123 (w), 1100 (w), 1045 (w), 1034 (w), 791 (m), 698 (s), and 648 (w).

(**CP3**) Anal. Calcd for [CuI­(C_6_H_7_N)]_n_: C, 25.41; H, 2.49; N, 4.94; found: C, 25.46; H, 2.57; N, 4.94. IR (cm^–1^): 1611 (m), 1495 (m), 1420 (m), 1382 (w), 1329 (w), 1221 (m), 1208 (m), 1067 (w), 1013 (m), 805 (s), 718 (m), and 672 (w).

(**CP4**) Anal. Calcd for [CuI­(C_6_H_8_N_2_)]_n_: C, 24.14; H, 2.70; N, 9.38; found: C, 24.05; H, 2.79; N, 9.27. IR (cm^–1^): 3406 (w), 3314 (w), 3209 (w), 1630 (s), 1592 (m), 1556 (m), 1488 (w), 1436­(m), 1376 (w), 1311 (m), 1242 (w), 1182 (w), 1000 (w), 952 (m), 799 (s), 770 (w), 746 (w), and 638 (w).

(**CP5**) Anal. Calcd for [CuI­(C_6_H_6_NCl)]_n_: C, 22.66; H, 1.90; N, 4.40; found: C, 22.48; H, 1.86; N, 4.20. IR (cm^–1^): 1596 (w), 1541 (w), 1472 (w), 1380 (w), 1284 (w), 1220 (w), 1132 (m), 1084 (m), 1004 (w), 880 (w), 828 (s), and 720 (w).

PXRD and IR of the CPs are in Figures S1 and S2.

#### 2.2.2 **CP3**–**CP5**, Single Crystals

Single crystals of compounds **CP4** and **CP5** were obtained by a slow diffusion or layering technique at room temperature. In this respect, three phases were prepared and carefully deposited one on top of the other in a sample tube. The upper phase is a solution of 4-methylpyridine (47 mg, 0.5 mmol), 2-amino-4-methylpyridine (54 mg, 0.5 mmol), or 2-chloro-4-methylpyridine (64 mg, 0.5 mmol) dissolved in 2 mL of ethanol, the middle phase is pure ethanol (1 mL), and the lower phase is a solution of CuI (95 mg, 0.5 mmol) in 2 mL of concentrated aqueous KI solution. Both sample tubes were capped with Parafilm and allowed to stand in a vertical position. After 24 h, suitable colorless needle-shaped crystals were obtained at the interface between the EtOH layer and the concentrated aqueous KI solution layer. These crystals were useful for single-crystal X-ray diffraction studies.

### 2.3 Preparation of TiO_2_/CP (CP1-CP5) Catalysts

A 20 mg portion of a 50:50 mixture of TiO_2_ and the corresponding CP as cocatalyst was weighed. They were ground for 5 min in an agate mortar until a homogeneous mixture was obtained. It was then suspended in 5 mL of a 0.5 M potassium bicarbonate solution with a pH of 8.5 and sonicated for 15 min to break up the agglomerates. Finally, this suspension was used to carry out the photoreduction tests.

### 2.4 Methods and Equipment

Infrared spectra (FTIR-ATR) were recorded on a PerkinElmer spectrophotometer equipped with a MIRacle universal attenuated total reflectance accessory (ATR).

Powder X-ray Diffraction (PXRD) data were collected on a Rigaku Miniflex 600 HyPix-400 MF 2D together with a 600W X-ray source diffractometer with geometry θ/2θ (Cu–Kα radiation; λ = 1.5418 Å). The samples were scanned over a range of 3° to 50° (θ/2θ), using an angular increment of 0.03° and a counting time of 10° per minute.

Elemental analysis (EA) measurements were carried out by using a LECO CHNS-93217 elemental analyzer.

Single-crystal X-ray diffraction (SCXRD): single-crystal X-ray diffraction data for structure determination were collected on an XtaLAB Synergy, single-source at home/near, HyPix diffractometer (λ Mo Kα = 0.71073 Å at 295 K). Data reduction was done with the CrysAlisPro program (Rigaku)^54^ and refined by full-matrix least-squares on *F* ^2^, including all reflections (SHELXL).^55^ All calculations for these structures were performed using the OLEX2 crystallographic software package programs.^56^ Details of the structure determination and refinement of all compounds are summarized in Table S1 of the Supporting Information.

Diffuse Reflectance (DRS): measurements were carried out on a Varian Cary 500 spectrophotometer fitted with an integrating sphere accessory. The resulting diffuse reflectance data were transformed using the Kubelka–Munk function,^57^ which enables the conversion of reflectance values to an equivalent absorbance unit. This transformation provides a basis for plotting the Tauc expression,^58^ often used to analyze optical band gaps.^59^

Thermal analysis (TGA) was performed on a METTLER TOLEDO TGA/SDTA851 thermal analyzer in synthetic air (80% N_2_, 20% O_2_) flux of 50 cm^3^ min^–1^, from room temperature to 800 °C with a heating rate of 5 °C min^–1^ and a sample size of about 10–20 mg per run.

Scanning electron microscopy (SEM) images were acquired on a Hitachi S-3000N SEM-EDX electron microscope, operating at an accelerating voltage of 5.0 kV under a chamber pressure of 10^–9^ Pa. Prior to imaging, the samples were coated with a thin chromium layer at a pressure of 1 × 10^–3^ Pa.

N_2_ and CO_2_ adsorption isotherms were measured using a Micromeritics 3Flex volumetric instrument under static adsorption conditions. Samples were previously activated at 50 °C overnight and outgassed to 10^–6^ bar. According to IUPAC recommendations, the specific surface area by the BET method can be estimated in the range of *P*/*P*_0_ = 0.05 to 0.30 for nonporous and macroporous solids (type II isotherms), as well as for mesoporous materials (type IV isotherms).^60^ However, for microporous materials (type I isotherms), this range may be affected; in such cases, it is appropriate to use lower relative pressures following the Rouquerol criteria.^61^ These criteria require that the BET constant “*C*” is positive, that the *n*(1 – *P*/*P*_0_) term increases continuously with *P*/*P*_0_ over the chosen range, and that the value of *P*/*P*_0_ that determines the monolayer capacity is within the range. In general, for type I isotherms, the applicable range is usually *P*/*P*_0_ < 0.08.

X-ray photoelectron spectroscopy (XPS) measurements were performed on a Versaprobe III AD Physical Electronics (ULVAC) system with a monochromatic Al Kα (1486.7 eV) radiation source. An initial analysis was carried out to determine the elements present (wide scan: step energy 0.2 eV, pass energy 224 eV) and detailed analysis of the detected elements (detail scan: step energy 0.05 eV, pass energy 27 eV, time per step 20 ms (pass energy 13 eV and step energy 0.025 eV for BV region)) was performed with an electron output angle of 45°. The spectrometer was previously calibrated with Ag (Ag 3d_5/2_, 368.26 eV). The XPS data were processed using CasaXPS 2.3.26 software.

CO_2_ photoreduction experiments were carried out using a mixture of TiO_2_/CP in different proportions, by manual grinding in a mortar agata for 5 min. Additionally, a PhotoRedOX Box reactor equipped with a monochromatic UV lamp (Hepatochem, λ_exc = 365 nm) with 10 W power was used. For the reaction, 20 mg of a heterogeneous catalyst mixture was placed in a Schlenk tube containing 5 mL of 0.5 M potassium bicarbonate solution at a pH of 8.5. The tube was sealed with a septum crossed by a syringe connected to a CO_2_ cylinder, with a flow rate of approximately 100 mL·min^–1^. To the side arm of the Schlenk tube was attached an 8 mm internal diameter conduit to evacuate the carrier gas. A 15 cm hypodermic needle was attached to the opposite end and inserted into the septum of a test tube containing 6 mL of distilled water. This test tube was immersed in an ice/water bath at a temperature between 0 and 4 °C. The long needle ensured bubbling of the gas to the bottom. Another needle, 3 cm long and located above the water level, was used as a final escape route for the carrier gas. With this arrangement, the produced alcohol circulates through the 15 cm needle into the test tube, which acts as a trap for condensable species, while the carrier gas exits through the 3 cm needle (Figure 4). The experiment is conducted for 10 h in 5 cycles of 2 h each. The first three trials were carried out continuously, while the remaining two took place after 12 h of rest. The liquid phase retained in the cold trap was analyzed with a gas chromatograph (Shimadzu Nexis GC-2030) equipped with a 30 m Shimadzu SH-Wax column (He as a carrier gas, flow rate 1.54 mL.min^–1^) and a dielectric barrier discharge ionization detector (BID). The operating temperature was set at 35 °C, and injections (5 μL in splitless mode) were performed in triplicate to assess reproducibility. A calibration curve was prepared for methanol and ethanol in the range of 0 to 100 ppm, and limits of detection and quantification were established from the signal-to-noise ratio. Any measurement below these values was discarded.

### Theoretical Calculations

All the simulations were performed in the frame of the density functional theory (DFT) with the Vienna ab initio simulation package (VASP),^62^ which considers periodic boundary conditions for a proper description of the materials. The electronic structure was expanded in a plane-wave basis with an energy cutoff of 400 eV, and the interaction between electrons and nuclei was described using the projected augmented wave (PAW) pseudopotentials, as provided by the VASP source. Reciprocal space was sampled with the Monkhorst–Pack scheme using a grid of 3–5–1 points. For geometry optimizations, the optPBE functional^63^ was used. Convergence threshold for electronic energies and forces were 10^–5^ eV and 0.01 eV/Å respectively. On top of the optimized structures, single-point calculations with the hybrid HSE06 functional^64^ were performed to obtain more accurate band gaps.

## 3 Results and Discussion

### 3.1 Chemical and Morphological Characterization

The synthesis of these CPs as suitable single crystals useful for SCXRD implies the diffusion at room temperature between the corresponding ligand (pyridine (**CP1**), 3-methylpyridine (**CP2**), 4-methylpyridine (**CP3**), 2-amino-4-methylpyridine (**CP4**), and 2-chloro-4-methylpyridine (**CP5**)) dissolved in ethanol, and CuI dissolved in a saturated aqueous solution of KI. The crystal structures of **CP1**, **CP2**, and **CP3** have been previously reported,^52,53,65^ and the crystallographic data for **CP4** and **CP5** are provided in Table S1. All of them present a one-dimensional structure featuring a ladder-like motif along the Cu­(I)-I chains, in which copper­(I) centers are interconnected via μ_3_-I bridges (Figure 1).^66^ The copper­(I) binds to the nitrogen of a pyridine ligand, adopting a local tetrahedral geometry. The major change between these compounds relies on whether the pyridinic ligand presents a substitution in the ortho positions. **CP1–3**, which do not have ortho substituents, provide very similar structural features for their 1D coordination polymer, as can be seen in Table S2. However, **CP4** and **CP5** with ortho substituents have a less regular 1D coordination polymer in which short and long Cu···Cu distances alternate (**CP4**: 2.778 and 3.517 Å; **CP5**: 2.696 and 3.517 Å). The shortest Cu···Cu distances are below the sum of the van der Waals radii, approximately 2.8 Å, and indicate a meaningful interaction between these Cu centers that can play a significant role in their photocatalytic properties.^67,68^ There are also changes in the dihedral angle between consecutive Cu_2_I_2_ planar segments that are now more acute, and also, the pyridinic ring is more twisted with respect to the step of the ladder. Another interesting feature related to **CP4** is the N–H···I hydrogen bond taking place within the ladder chain and probably related to the latter, its short Cu–N bond distance, which is the shortest one within this family of compounds (Figure 1).

**Figure 1:**
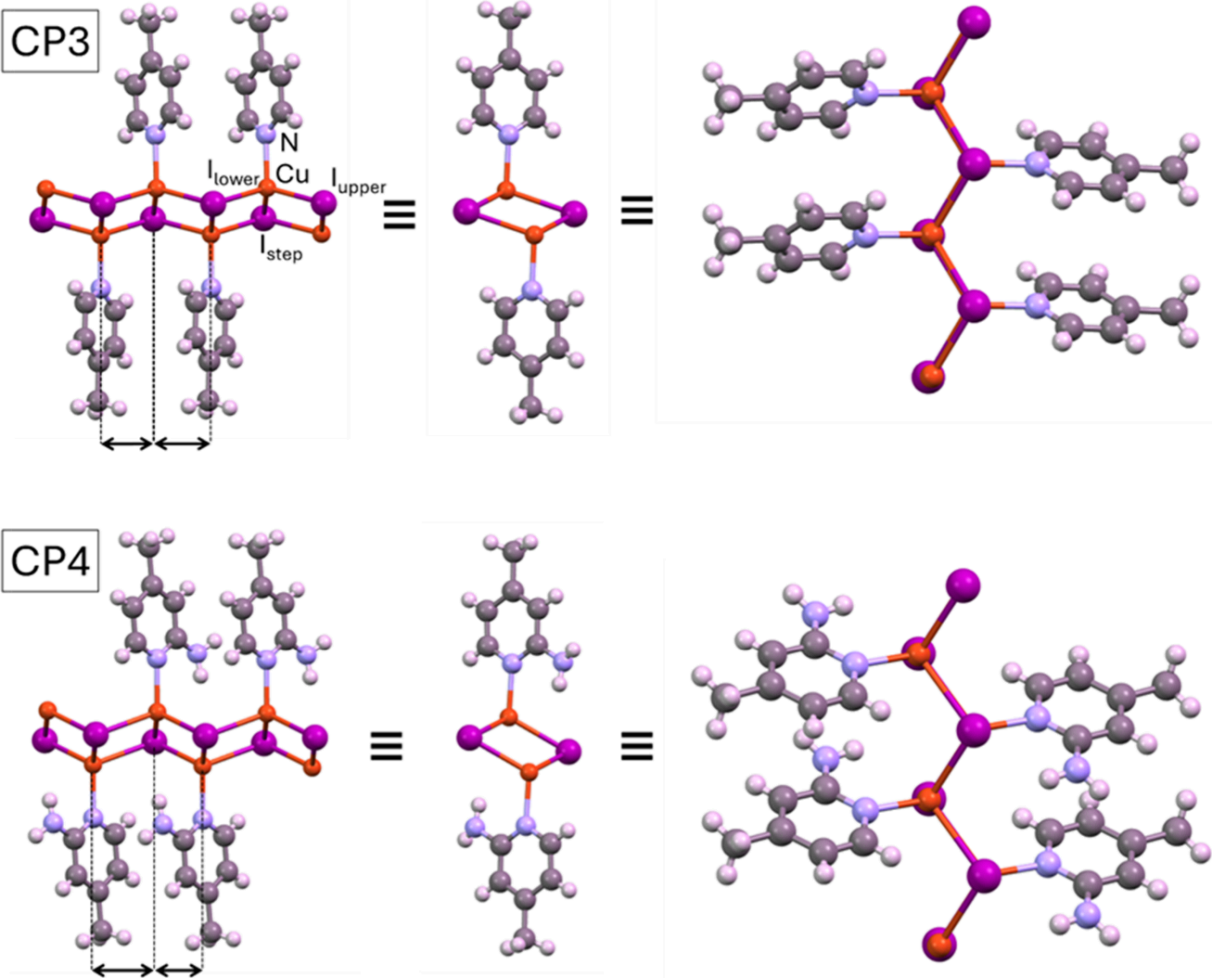
Views of the 1D CPs of **CP3** and **CP4** highlighting the differences in the alternation pattern.

The **CP1–3** coordination chains are held together by van der Waals-type interactions without evidence of π-stacking interaction between the aromatic rings of the organic ligand. **CP5** presents chloro···aromatic interactions^69^ within the chain, but the interactions holding the chains together are again van der Waals type. **CP4** presents stronger supramolecular interactions with hydrogen bonds (N–H···N: 3.28 Å, 158°) being established between the amino groups of adjacent chains and involving an intrachain hydrogen bond involving the amino group and the bridging iodide anions (N–H···I: 3.80 Å, 132°), providing additional stabilization to the crystal structure (Figure 2b). In fact, TGA data on these CPs indicate that **CP4** exhibits the highest thermal stability (ca. 180 °C), whereas **CP1–3** are stable up to 115–140 °C and **CP5** is only stable up to 100 °C (Figure S4). Ultimately, these secondary interactions determine the final architecture, shifting from packing dominated by van der Waals interactions in **CP1–3** and **CP5** to a more compact and directional arrangement in **CP4**, where hydrogen bonds between chains play a key role in stabilizing the structure.

**Figure 2:**
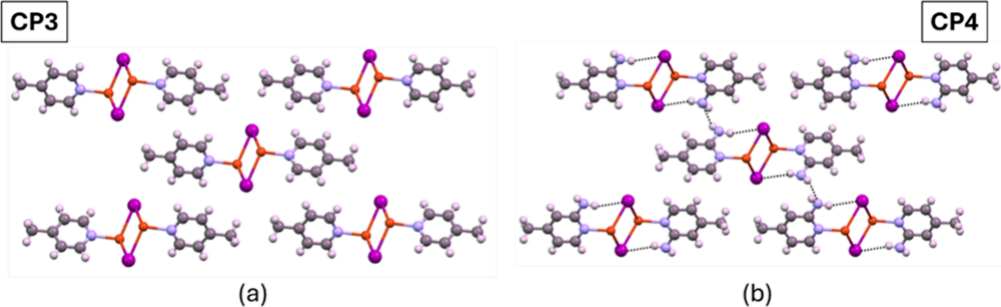
Crystal packing of compounds **CP3** (a) and **CP4** (b) viewed along the chain propagation direction. Dashed lines indicate hydrogen bonds.

These CPs can also be synthesized as microcrystalline powders within minutes in a one-step reaction at room temperature using water as the only solvent and stoichiometric amounts of the building blocks. This new synthesis route is easily scalable and fulfills the green chemistry criteria. The reaction yields ranged from 40% for **CP5** to 75% for **CP3**. Elemental analysis, FTIR-ATR, and PXRD confirm that these powder samples correspond to **CP1–5** (Figures S1 and S2). The most representative IR signals include ν­(C=C + C=N) stretching bands between 1650 and 1550 cm^–1^, and δ­(C–H) bending bands between 1450 and 1350 cm^–1^. More specific bands correspond to the stretching ν­(C–C) in **CP2–5** in the range 785–880 cm^–1^, ν­(C–N) in **CP4** at 1245 cm^–1^, and ν­(C–Cl) in **CP5** as a very intense band at 825 cm^–1^. Likewise, in **CP4**, v­(N–H) stretching bands are identified in the range of 3200 to 3400 cm^–1^. SEM micrographs of the microcrystalline powder (Figure 3a and Figure S3) reveal a microrod morphology, in all cases, consistent with the 1D crystalline structures of these materials. Statistical analysis of the rod lengths yields average values ± standard deviation of: **CP1** 21 ± 8 μm, **CP2** 20 ± 8 μm, **CP3** 18 ± 8 μm, **CP4** 16 ± 6 μm, and **CP5** 19 ± 9 μm.

**Figure 3:**
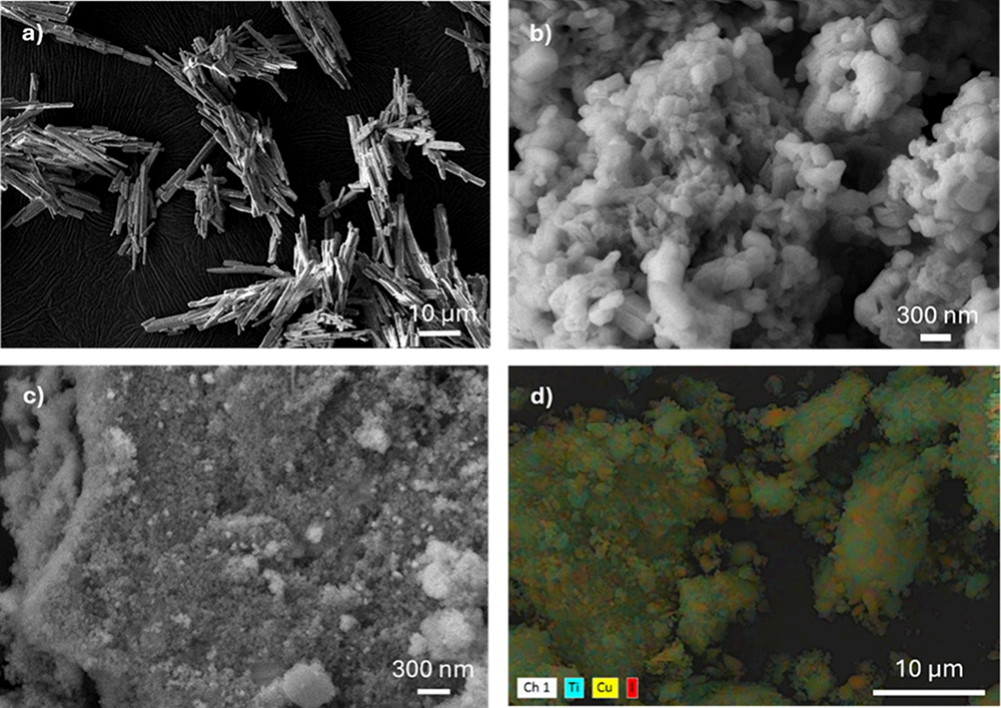
SEM micrographs of the CP4: (a) microcrystals before grinding, (b) after grinding, (c) ground TiO_2_@CP4, and (d) ground TiO_2_@CP4 SEM-EDX.

### 3.2 CO_2_ Photoreduction Experiments

Initial CO_2_ photoreduction experiments did not provide evidence of alcohol production for any of these CPs. Therefore, it was decided to employ a mixture of TiO_2_ nanoparticles in combination with ground CPs to create heterojunctions that could boost the CO_2_ reduction capability. SEM micrographs reveal that the grinding procedure transforms the initial rod-like particles of CPs (e.g., **CP4** with an aspect ratio of 5–20 × 1–2 μm) into more isotropic granules with sizes reduced to 100–300 nm (Figure 3b and Figure S5), maintaining their initial crystalline phase, as is confirmed by FTIR-ATR and PXRD analyses (Figures S6 and S7). Furthermore, mechanical grinding with TiO_2_ nanoparticles facilitates their uniform distribution across the surface of the CPs. This is evidenced by SEM-EDX elemental mapping, which reveals extensive regions where Ti and Cu signals overlap, indicating a relatively homogeneous dispersion of TiO_2_ nanoparticles (∼20 nm) (Figure S8) coating the larger CP particles (Figure 3c,d, Figures S9 and S10). Complementary FTIR-ATR and PXRD analyses further confirm that the chemical integrity of the coordination polymer is preserved after mixing with anatase (**TiO** **_2_** **@CP**) (Figures S11 and S12).

DRS spectra were recorded before and after grinding to estimate the optical band gaps via the Kubelka–Munk function (Table 1, Figures S13–S16). There is a slight increase in the band gap after grinding, probably related to the reduction of the particle size into the submicrometric regime, along with probably an increased amount of defects.^70^

**Table 1:** Band Gap Values (in eV) of CPs and TiO_2_, before and after Grinding

	CP1	CP2	CP3	CP4	CP5	TiO_2_
before grinding	2.91	2.94	2.37	3.50	2.96	3.36
after grinding	3.01	3.00	3.11	3.55	3.01	3.36

To ensure reliable and comparable results, the photocatalytic CO_2_ reduction process involves multiple experiments conducted under identical conditions (reactor, experimental setup, light source, and reaction parameters).^71^ An arbitrary 50:50 wt % initial Ti/CP ratio (**TiO** **_2_** **@50%CP**) is used for the photocatalytic studies. The results obtained for the different photocatalytic systems are compared, and the one exhibiting the highest chemical stability, determined by PXRD and FTIR, and the highest alcohol production is selected for an optimization of the cocatalyst mixture ratio.

To perform the photocatalytic experiments, the catalyst mixture is placed in a Schlenk flask containing an aqueous potassium bicarbonate solution and sealed with a septum. CO_2_ is continuously bubbled through the system at ∼25 °C (Figure 4) while irradiated with a UV lamp (λ = 365 nm). The gas outlet is directed to a cold-water trap to condense the produced alcohols. The experiment was carried out over 10 h over 5 cycles of 2 h. The first three cycles were carried out continuously, while the remaining two took place after 12 h of rest.

**Figure 4:**
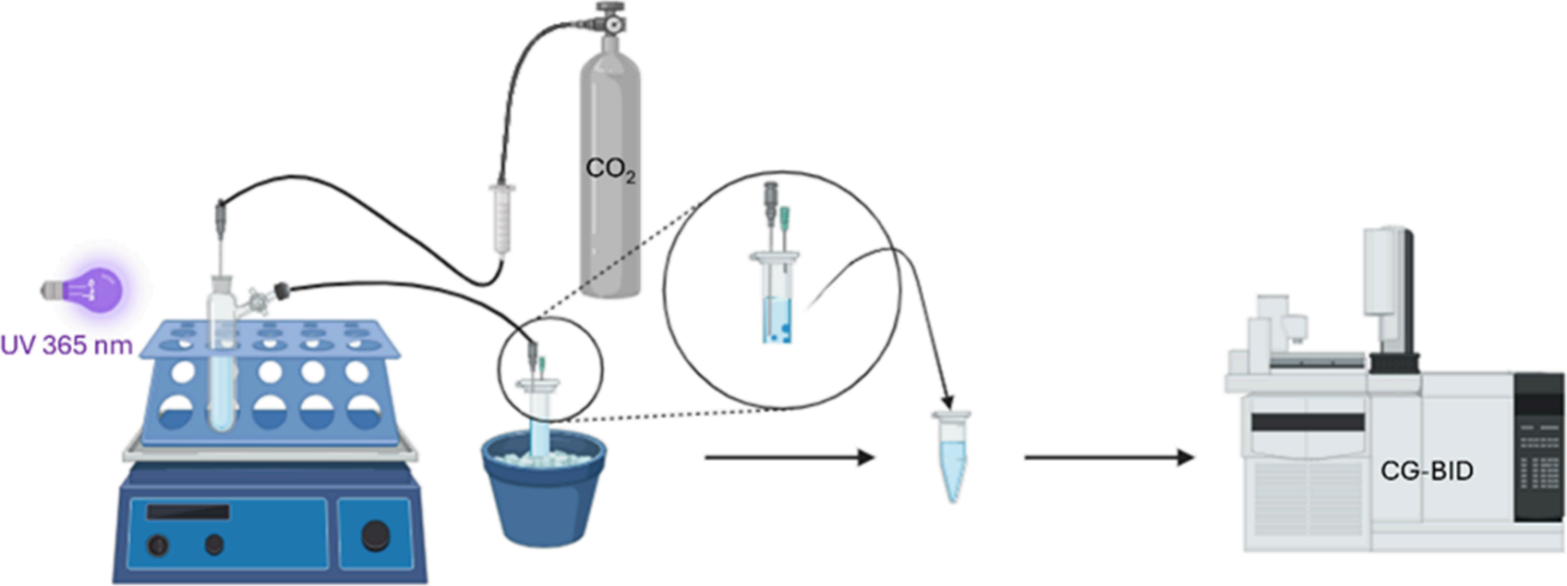
Scheme of experimental configuration employed for the CO_2_ photoreduction to alcohol studies (see detailed information in the “Experimental Section” part).

First, the photocatalytic activity of pure anatase is evaluated. This is followed by tests using CuO and Cu_2_O as cocatalysts with TiO_2_,^23,24^ as these materials have shown promising results in CO_2_ photoreduction to alcohols and can be useful as references in our measurement conditions. Given that the studied CPs contain CuI chains, the photocatalytic performance of anatase combined with CuI (**TiO** **_2_** **@CuI**) is also investigated. Finally, the CO_2_ photoreduction capacity of anatase mixed with the synthesized CPs is assessed.

The introduction of 50 wt % Cu_2_O as a cocatalyst (**TiO** **_2_** **@50%Cu** **_2_** **O**) significantly enhances methanol and ethanol production compared to pure anatase, but it is the **TiO** **_2_** **@3%CuO** catalytic mixture which outstands among the reference cocatalytic systems, reaching 318 μg·g_cat_^–1^·h^–1^ of methanol selectively (Figure 5). The **TiO** **_2_** **@50%CuI** system achieved a methanol yield of 84 μg·g_cat_^–1^·h^–1^, also with 100% selectivity (Figure 5). All **TiO** **_2_** **@50%CPs** systems produce, at some point, amounts of alcohol rivaling those of **TiO** **_2_** **@3%CuO** (Figure 5). The stability of the photocatalytic systems was studied upon five consecutive CO_2_ photoreduction cycles, each lasting 2 h. The alcohol production along the 10 h run is quite irregular except for **CP4**. The total accumulated alcohol production ranges from 2640 μg·g^–1^ for **TiO** **_2_** **@50%CP2** to 3640 μg·g^–1^ for **TiO** **_2_** **@50%CP4**, with methanol selectivity varying from 50% for **TiO** **_2_** **@50%CP5** to 72% for **TiO** **_2_** **@50%CP4** (Figure 5). In addition, there is a decay in alcohol production over time for **TiO** **_2_** **@50%CP1–3** (Figure 5). **TiO** **_2_** **@50%CP4** shows no clear evidence of decay (Figure 5).

**Figure 5:**
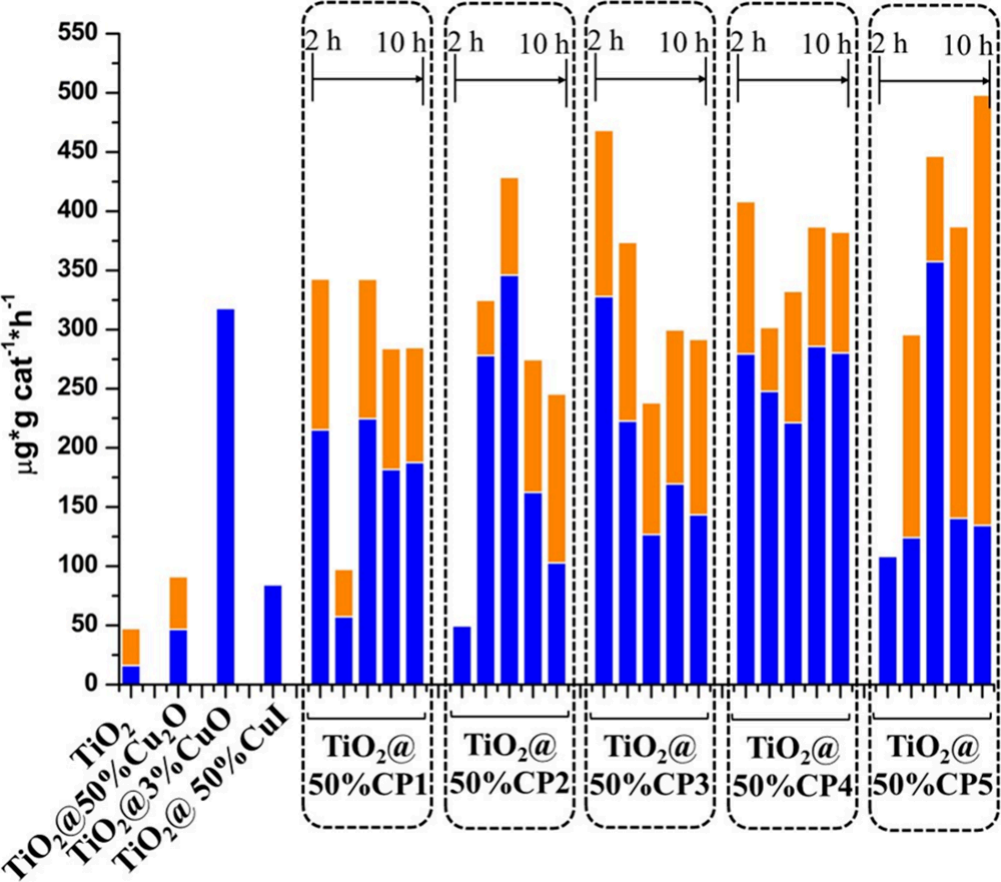
Alcohol production rates for TiO_2_ and **TiO_2_@50%CP** systems. Methanol and ethanol production are presented in blue and orange color, respectively.

After the photocatalysis experiments, the particles of the cocatalyst mixture were recovered and analyzed by FTIR-ATR spectroscopy, PXRD, SEM, and SEM-EDX microscopy (Figures S17–S20). The FTIR-ATR spectra do not provide information on the inorganic cocatalyst (TiO_2_), but they do allow characterization of the CP cocatalyst. **CP1**-**CP4** do not undergo appreciable alterations after the photocatalytic process, with the characteristic signals of the pyridinic ligand being retained (Figure S17). However, the FTIR-ATR of **CP5** shows the disappearance of the ligand bands, indicating its transformation after 10 h of photocatalytic reaction (Figure S17). The PXRD obtained after the CO_2_ photoreduction process shows no variation in the patterns of **TiO** **_2_** **@50%CP1-CP4** (Figure S18); however, in **TiO** **_2_** **@50%CP5**, the **CP5**-associated diffraction peaks disappear (Figure S18). This suggests that the crystal structures of **CP1**-**CP4** are unchanged, while **CP5** does not withstand the photocatalytic conditions that release the organic ligand.

SEM and SEM-EDX images clearly indicate that the homogeneity of the initial **TiO** **_2_** **@50%CP** catalytic mixtures is only retained, after the photoreduction reaction, for **TiO** **_2_** **@50%CP4** (Figure 6 and Figures S19 and S20). The remaining mixtures, **TiO** **_2_** **@50%CP1–3**, show growth of the CP particles into the micrometric regime again with the segregation of the TiO_2_ nanoparticles. This growth of the **CP1–3** particles seems to be due to a solubilization/recrystallization process, and this, although not clearly seen in the 10 h photocatalytic run, could have a negative impact on the alcohol production because of the surface area reduction. The apparent lower solubility of **CP4** could be related to the presence of stronger supramolecular interactions (N–H···N hydrogen bonds) holding the coordination chains together, which limits particle growth and improves the structural and chemical stability. Although SEM-EDX images of the solid products may not accurately reflect the distribution within the reactor, where the mixture is suspended in a stirred aqueous solution, the analysis of **TiO** **_2_** **@50%CP4** after photocatalysis by this technique reveals localized regions with a slightly more heterogeneous distribution between TiO_2_ and **CP4**, possibly indicating partial detachment of TiO_2_ from the CP surface. However, this effect appears to be minimal, as **TiO** **_2_** **@50%CP4** maintains consistent photocatalytic performance over time, exhibiting excellent recyclability, attributed to its strong interfacial stability and resistance to structural and chemical degradation. Moreover, the photocatalytic experiment consisted of an initial 6 h measurement, followed by a 12 h pause (overnight) and an additional 4 h run. The lack of significant variation in alcohol production during this period provides further confirmation of the robustness and recyclability of the photocatalytic system.

**Figure 6:**
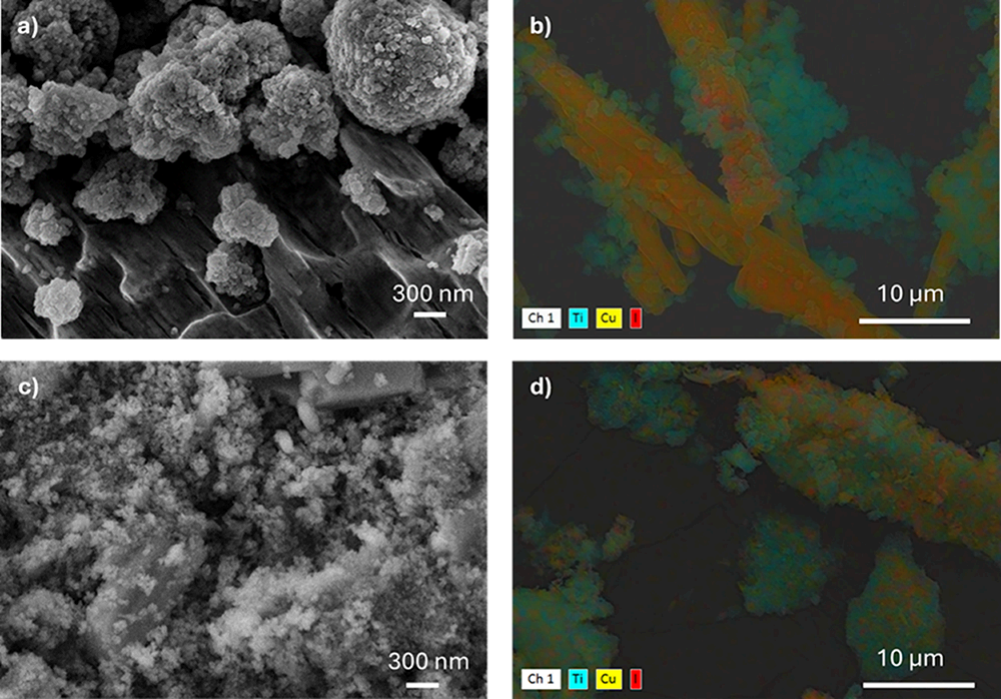
SEM and SEM-EDX micrographs of the **TiO_2_@50%CP1** (a, b) and **TiO_2_@50%CP4** (c, d), after 10 h of CO_2_ photoreduction.

The reason for the good performance of the **TiO** **_2_** **@50%CP** catalytic systems can be attributed to several reasons, but perhaps the two most important ones would be interaction with the CO_2_ molecule^72^ and the energy arrangement of the CB and VB in both cocatalysts. In fact, the photocatalytic reduction of CO_2_ on the surface of a semiconductor involves five fundamental steps: (1) CO_2_ adsorption, (2) light absorption, (3) charge separation and transfer, (4) surface redox reactions, and (5) desorption of the reaction products.^73^ Therefore, it is important to determine if the incorporation of CP cocatalysts enhances the CO_2_ adsorption, which can occur via the carbon atom, oxygen atoms, or a mixed mode.^74–76^ To this end, N_2_ and CO_2_ adsorption isotherms were measured at 77 and 273 K, respectively (Figures S21–S24). Table 2 lists the specific area values determined by the BET method together with the maximum adsorption capacity of CO_2_. The N_2_ isotherms of the CPs (Figure S22) present a type II profile and exhibit very low surface areas, confirming their nonporous character. In contrast, the isotherms corresponding to TiO_2_ and the **TiO** **_2_** **@50%CP** (Figures S21 and S23) are of type IV and show similar hysteresis loops, evidencing the presence of mesopores in both samples, probably coming from the interparticle space. The calculated BET surface area of TiO_2_ nanoparticles (83.3 m^2^/g) is diluted when mixed with CP particles. Interestingly, the BET surface area is more than halved with respect to TiO_2_, which can be understood as a sign of good adhesion between both types of particles, as shared surfaces will not be computed as an external surface. The mixtures with lower surface area values are **TiO** **_2_** **@50%CP3–4.** The CO_2_ adsorption capacity of **TiO** **_2_** **@50%CPs** is also reduced with respect to TiO_2_, but normalizing these values with respect to the surface area also provides an insight into the CO_2_ interaction with these CPs, with **TiO** **_2_** **@50%CP4** providing a higher CO_2_ capture per surface. Total alcohol production was also normalized with respect to the surface area; **TiO** **_2_** **@50%CP4** yielded a higher alcohol production per surface area.

**Table 2:** Textural Characterization of TiO_2_, **CP1–4**, and TiO_2_@50%**CP1–4**

	TiO_2_	CP1	CP2	CP3	CP4
*S*_BET_ (m^2^/g)^a^	83.3	5.16	2.45	4.29	2.69

mixture of TiO_2_/CP 50 wt %					
*S*_BET_ (m^2^·g^–1^)^a^	83.3	31.2	26.9	24.4	25.5
*C*_CO2_273 K (mmol·g^–1^)^b^	0.153	0.062	0.066	0.064	0.097
*C*_CO2_* 273 K (10^–3^ mmol·m^–2^)^c^	1.85	1.99	2.46	2.63	3.79
Γ (μg·g_cat_^–1^·h^–1^)^d^	47.4	270	264	333	363
Γ *(μg·m^–2^·h^–1^)^e^	0.569	8.67	9.85	13.6	14.3

a Surface area BET.

b CO_2_ adsorbed capacity.

c CO_2_ adsorbed capacity normalized to surface area.

d Average total alcohol production.

e Average total alcohol production normalized to surface area.

XPS studies were carried out to determine the processes taking place at the surface level of **TiO** **_2_** **@50%CP4** during the photocatalytic reaction. Spectra were recorded for pure **CP4** (Figure S25) and **TiO** **_2_** **@50%CP4** after the photoreduction reaction (Figure S26). The Ti^4+^ characteristic Ti 2p_3/2_ (458.2 eV) and Ti 2p_1/2_ (463.9 eV) signals agree with those reported for commercial pure TiO_2_ nanopowder (Degussa P-25) (Table S3).^77^ However, the characteristic signals of Cu and I, in **TiO** **_2_** **@50%CP4**, are displaced toward smaller binding energies (0.3 eV for copper and 0.6 eV for iodine) with respect to **CP4** (Figure 7a,b). These variations are attributed to a higher electron density on **CP4** particles due to the formation of heterojunctions with TiO_2_. The absence of characteristic Cu^2+^ satellite peaks at higher binding energies (BE) indicates that the copper is mostly in the Cu^+^ state, in agreement with what has been reported for similar systems.^78^ Two main components are identified in the C 1s spectrum, with bond energies of 284.6 and 286.0 eV, assignable to C–C/C–H and C–N bonds,^79^ respectively. Additionally, the **TiO** **_2_** **@50%CP4** sample exhibits a third component at 288.2 eV, compatible with N–C=O bonds, which confirms the presence of carbonyl species derived from CO_2_ adsorbed on the surface after the photoreduction test (Figure 7c).^80^ The N 1s spectrum shows a main peak at 399.2 eV in **CP4** and at a slightly lower binding energy of 398.8 eV in **TiO** **_2_** **@50%CP4**, which are characteristic of the pyridine nitrogen in the pyridine ring.^81^ In addition, a secondary component at 400.4 eV appears in **TiO** **_2_** **@50%CP4**, which is attributed to the interaction of pyridine with CO_2_ (Figure 7d).^82^ The O 1s region in **TiO** **_2_** **@50%CP4** is resolved into two components: a main peak at 529.4 eV, attributed to oxygen in the TiO_2_ lattice, and a second at 530.9 eV, corresponding to carboxylate groups.^83^

**Figure 7:**
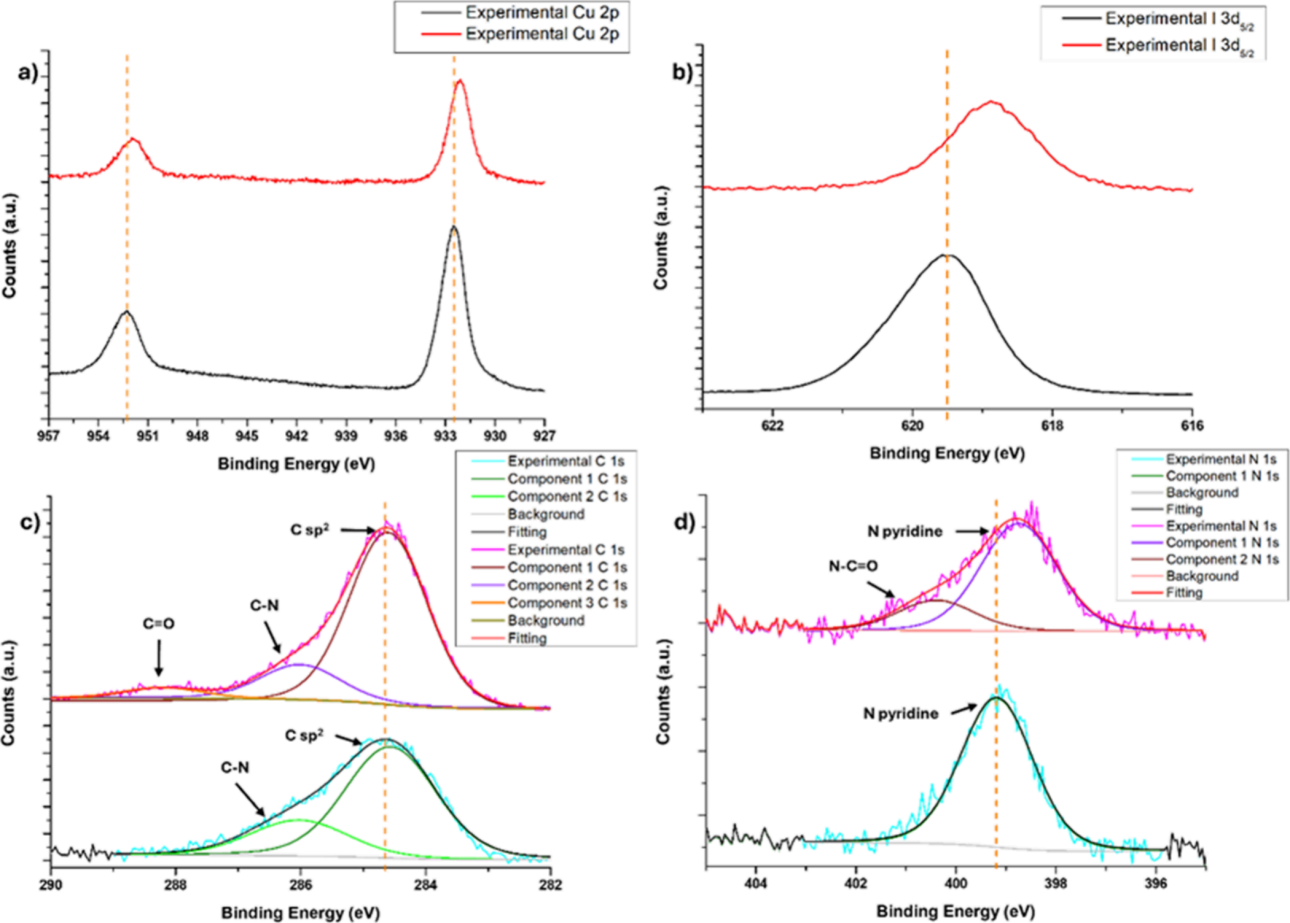
XPS spectra of **CP4** (bottom) and **TiO_2_@50%CP4** (top) in the Cu 2p region (a), I 3d region (b), C 1s region (c), and N 1s region (d).

XPS measurements in the low binding energy for TiO_2_ and **TiO** **_2_** **@50%CP4** give an insight into the relative energy of the valence band (VB) upper edge of TiO_2_ and **CP4**. These results indicate that the VBE (**CP4**) is 2.16 eV above that of TiO_2_ (Figure S27). Combining this information with the band gap values obtained from the DRS measurements allows us to depict the conduction band (CB) and the energy diagram present in the **TiO** **_2_** **@50%CP4** photocatalytic system (Figure 8), which strongly suggests the presence of a Z-scheme process that would explain the good performance of this mixture. Upon light irradiation, both TiO_2_ and **CP4** become photoexcited. The photogenerated electrons in the conduction band of TiO_2_ recombine with the photogenerated holes in the valence band of **CP4**, facilitating an effective charge transfer. This process results in a high reduction potential for the electrons in the conduction band of **CP4**, which drives the reduction of CO_2_ to alcohols, and a high oxidation potential for the holes in the valence band of TiO_2_, which promotes the oxidation of water to O_2_. Consequently, the system operates efficiently without the need for a sacrificial agent to sustain the photocatalytic cycle, making it a more sustainable and energy-efficient mechanism.

**Figure 8:**
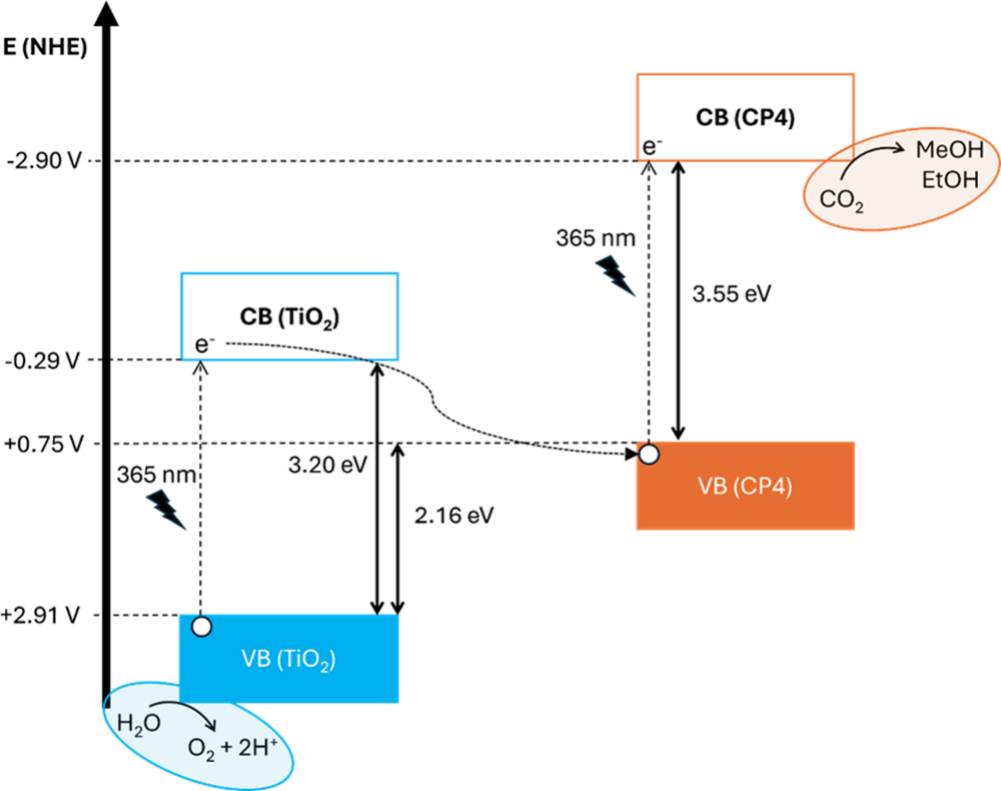
Normal hydrogen electrode (NHE) potential values of the TiO_2_ and **CP4** VB and CB edges based on the experimental DRS and XPS low binding energy measurements.

### 3.3 Optimization of the TiO_2_/CP4 Ratio

The alcohol production was maximized by optimization of the ratio between the two catalysts in the **TiO** **_2_** **@CP4** mixture. The photoreduction experiments were performed by varying the proportion of **CP4** (5, 25, 50, and 75% by mass), keeping the total mass constant at 20 mg (Figure 9). The results show a progression in the alcohol production when increasing the TiO_2_ proportion in the photocatalytic mixture, with values that change from null alcohol production for **TiO** **_2_** **@75%CP4** to 894 ± 80 μg·g_cat_^–1^·h^–1^ and 100% selectivity toward methanol for **TiO** **_2_** **@5%CP4**, with no evidence of decay during the 10 h run. This yield far exceeds the 318 μg·g_cat_^–1^·h^–1^ of methanol produced by the benchmark **TiO** **_2_** **@3%CuO** (Figure 4) and emphasizes the relevance of having abundant electron transfer from the TiO_2_ nanoparticles to **CP4**.

**Figure 9:**
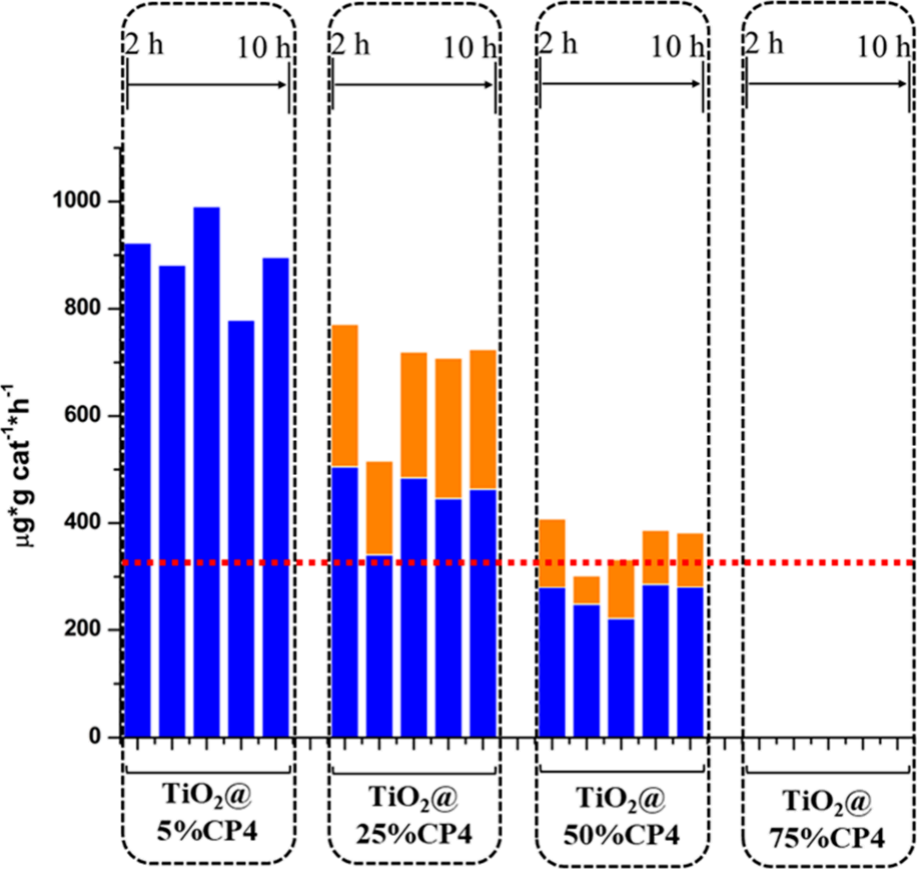
Alcohol production rates for **TiO_2_@CP4** at different cocatalyst ratios, keeping the total mass of the photocatalytic system at 20 mg. Five consecutive CO_2_ photoreduction cycles, each lasting 2 h, were performed for each **TiO_2_@CP4** system. Methanol and ethanol production are presented in blue and orange color, respectively. Red dotted lines correspond to the total alcohol production value of the reference mixture TiO_2_@3%CuO.

The selectivity toward methanol of **TiO** **_2_** **@5%CP4** contrasts with the methanol/ethanol mixtures obtained for the less TiO_2_-rich mixtures. The reason for this selectivity toward methanol, which cannot be attributed entirely to the chemical features of **CP4**, must be understood as a competition between the two reduction pathways, as illustrated in Figure 10. The mechanism that produces methanol is favored under highly reductive conditions, whereas the pathway that produces ethanol requires the formation of a C–C bond. This implies that the latter would be favored under conditions in which the initial intermediate single-carbon species have enough time to wait for a second CO_2_ molecule to be adsorbed at an adjacent position or to migrate along the surface of the cocatalyst, meet another single-carbon species, and evolve into two carbon species before being further reduced.^84,85^

**Figure 10:**
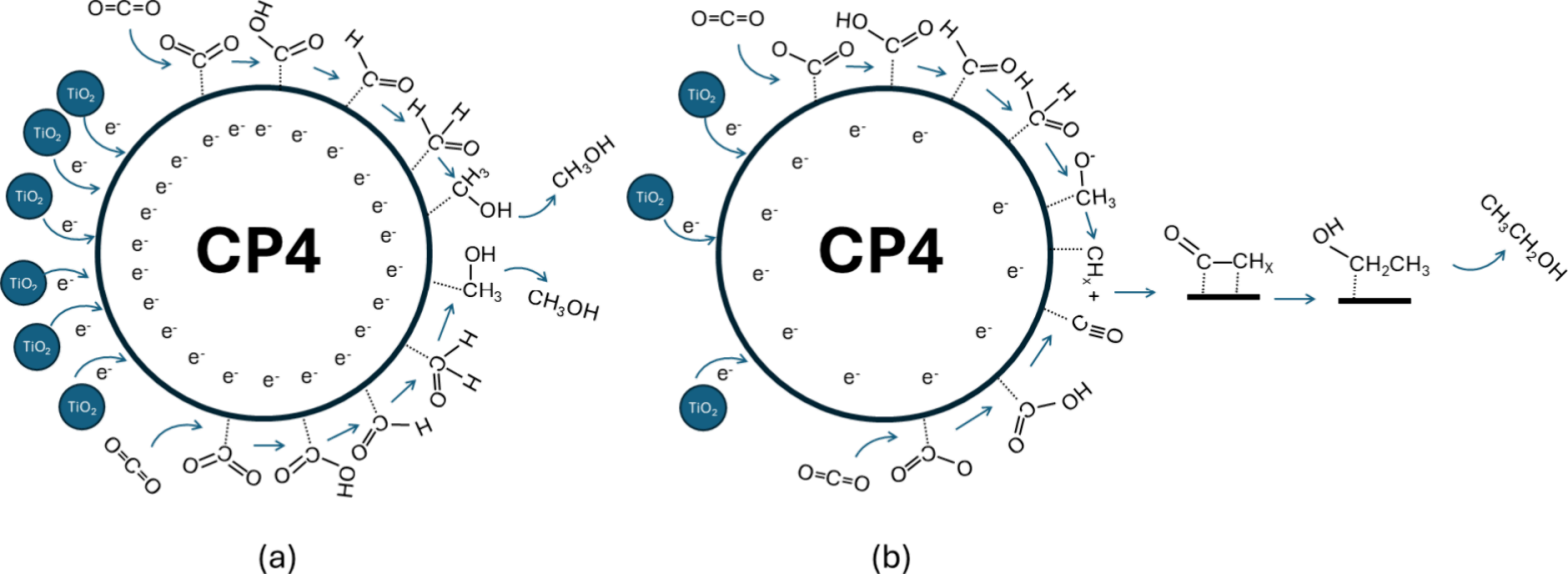
Proposed mechanisms for the different selectivities shown by (a) the catalytic mixture under TiO_2_-rich conditions (**TiO_2_@5%CP4**) and (b) more TiO_2_-poor conditions.

On the surface of **CP4**, methanol is produced through a multielectron, proton-coupled process in which adsorbed CO_2_ is initially activated (*CO_2_^–^/COOH) and then sequentially reduced via carbene/formaldehyde pathway [*CHOO^–^ → *CHOOH → *CHO → *CH_2_O → *CH_3_O → CH_3_OH].^2,73,86^ The high selectivity toward methanol in **TiO** **_2_** **@5%CP4** is attributed to the lower number of **CP4** active sites due to its reduced loading, combined with enhanced electron transfer from TiO_2_ nanoparticles to **CP4**. This reduces the residence time and stability of *CO-type intermediates on the surface, suppressing C–C coupling and favoring rapid proton-coupled electron transfer to the C_1_ products.

In contrast, ethanol formation proceeds through the coupling of two *CO intermediates: *CO species adsorbed on adjacent sites can migrate or encounter one another and undergo dimerization to form C_2_ intermediates, which then undergo multiple hydrogenation steps [1)*CO_2_ →*COOH → *CO/2) CHOO^–^ → *CHOOH → *CHO → *CH_2_O → *CH_3_O → *CH_*X*_ + *CO → *CH_*X*_CO → *CH_*X*_CHO → *CH_3_CH_2_O → C_2_H_5_OH].^87^ Under conditions where the surface coverage of *CO intermediates is higher or their residence time is longer, this coupling pathway becomes more favorable, resulting in increased ethanol production.

### 3.4 Computational Calculations

The XPS and DRS provided insight into the electronic structure of **CP4** was complemented with theoretical Density Functional Theory (DFT) calculations (see computational details in the “Experimental Section” part). These calculations provide not only approximate values of the VBE/CBE and the band gap energies, which can be compared to the experimental ones, but also the orbitals contributing to the energy levels located at these band edges. The procedure implies an optimization of the crystal structure of **CP1–4**, which produced deviations lower than 4% in the lattice constants with respect to the crystallographic ones. On top of these optimized cells, the projected density of states (PDOS) were computed, obtaining the contributions of the different angular momenta of each atom (Figure 11 and Figure S28).

**Figure 11:**
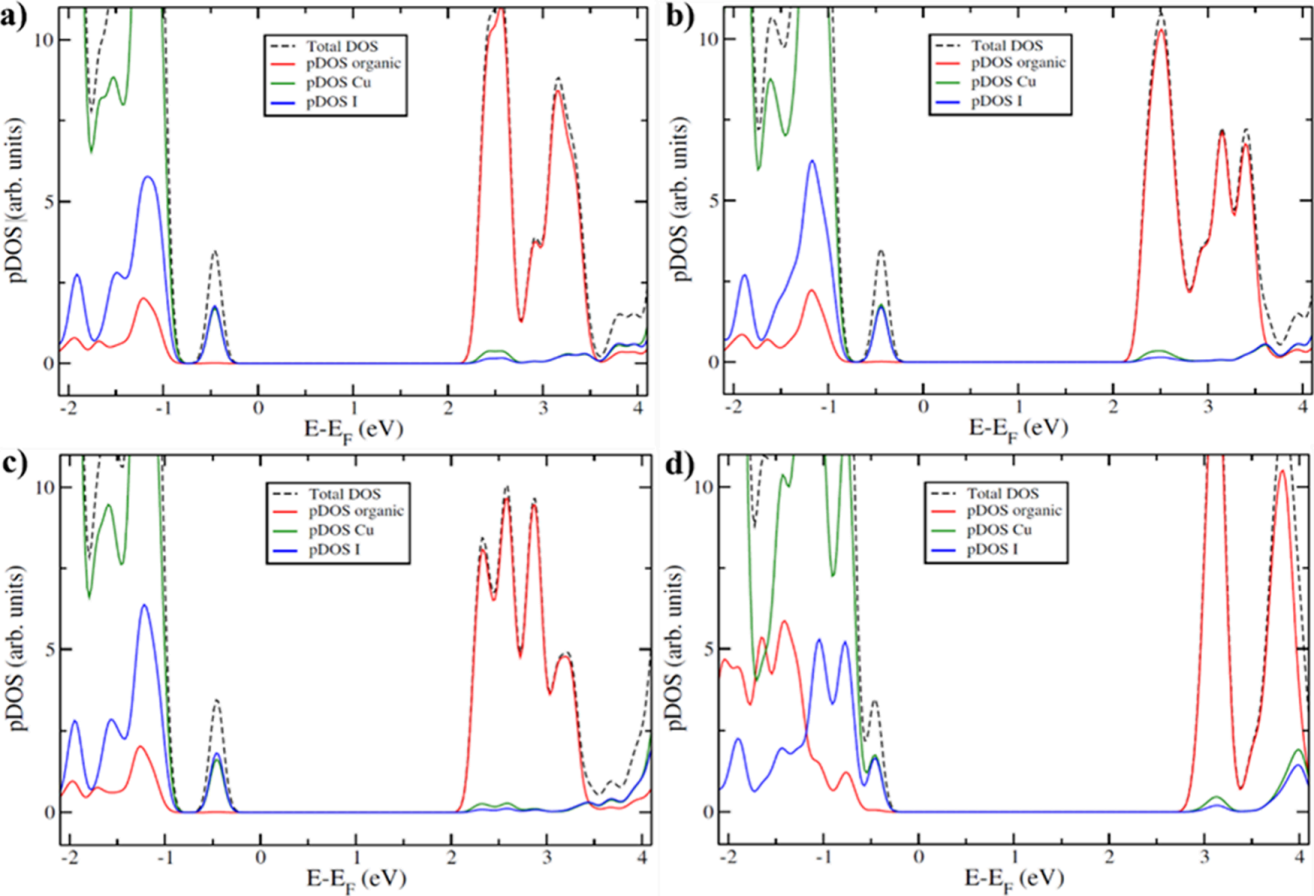
Density of states of (a) **CP1**, (b) **CP2**, (c) **CP3**, and (d) **CP4** with projection on the ligand atoms and on the Cu and I unit atoms.

In all of the computed CPs, the valence band energy (VBE) is largely located on the Cu and I atoms. However, the conductive band energy (CBE) is located mainly on the organic ligand atoms, pointing to a metal–ligand charge transfer transition. The computed band gap is 2.8 eV for **CP1** and **CP2**, 2.7 eV for **CP3**, and 3.4 eV for **CP4**, in good agreement with the experimentally measured values for the nongrinded CPs (Table 1). However, to explain the favorable effect of heterogeneous mixing between TiO_2_ and the CPs on the CO_2_ photoreduction, the relative positions of the VB and CB band edges of the cocatalysts used need to be considered. The absolute CB and VB edge energy values, referred to the vacuum, corresponding to the CPs have been calculated for all CPs, TiO_2_, and CuI. The latter ones establish the accuracy of the theoretical method. To this, we have calculated the difference between the Fermi level and the asymptotic (Hartree) potential in vacuum (Figure S29), which allows us to determine the work function of the material (Table S4). The remaining data for other cocatalysts have been extracted from the literature (Table S5).^88,89^ These energy levels translated into NHE potentials, together with the reduction potentials of CO_2_ to methanol and ethanol at pH 7.35, which corresponds to the value reached when bubbling CO_2_ in a 0.5 M potassium bicarbonate solution, are presented in Figure 12. There is a significant mismatch between the DFT theoretical and experimental band edge values, but the same trend observed on the experimental data is reproduced. TiO_2_ anatase provides the deepest energy levels, CuI raises these values probably because of the most reduced nature of its components, and the incorporation of the pyridines in the CuI increases these levels even more, approaching their VBE of CPs to the CBE of TiO_2_. The VBE of CPs gets closer to the CBE of TiO_2_, reinforcing a Z-scheme mechanism that provides efficient electron–hole spatial separation (Figure 8).

**Figure 12:**
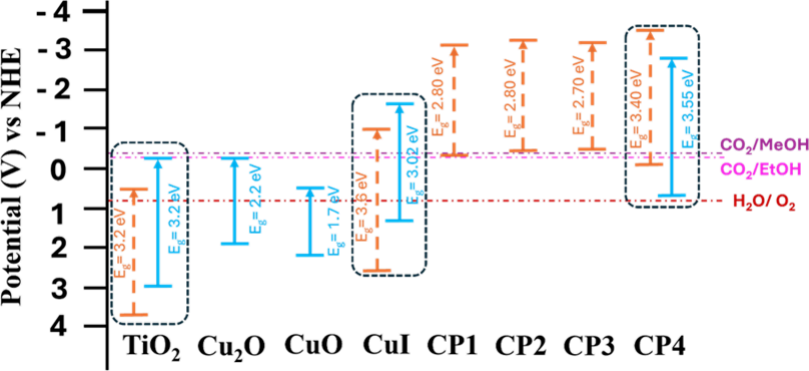
Energy values of the CB and VB edges of the photocatalysts, as reported in the literature and calculated for each CP and selected inorganic cocatalysts. Orange values correspond to DFT provided values (Table S4), and blue values are derived from the literature and, in the case of **CP4**, from experimental DRS and XPS low binding energy measurements (Table S5).

Theoretical calculations also allow the study of CO_2_ adsorption, which points to a higher (more negative, thus more favorable) adsorption energy in **CP2–4**, which are the compounds with substituents in the pyridine ring (Figure 13). The presence of these groups increases the van der Waals-type interactions that the CO_2_ molecule can establish with the surface of the CP particles, although the preferential adsorption site is close to the iodine atom of the Cu­(I)-I unit due to its higher electron density. Specifically, **CP3** and **CP4** show the highest theoretical affinities for CO_2_ (Figure 13). Although computational models do not differentiate between them, already described CO_2_ experimental adsorption isotherms confirm that **CP4** adsorbs significantly more CO_2_ than the other compounds (Figure S24).

**Figure 13:**
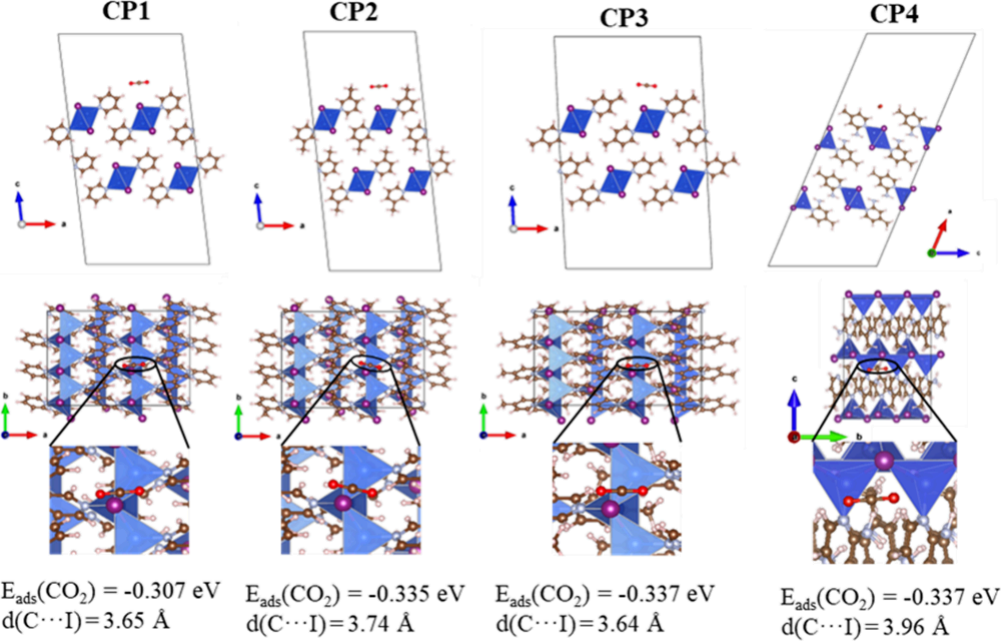
Preferred CO_2_ adsorption site on the most stable surface of each CP, computed adsorption energy values, and the C_CO2_···I distance.

This difference is attributed to the presence of an amino group at position 2 of the pyridine ring, adjacent to the Cu­(I)-I chain, which locally increases the electron density of the iodine atom and thereby enhances the CO_2_ interaction. This increase in electron density has also been observed in the XPS results, where iodine exhibits a lower BE in **TiO** **_2_** **@50%CP4** (Table S3 and Figure S26) compared to pure **CP4** (Table S3 and Figure S25). This suggests a mechanism in which CO_2_ is attracted to and stabilized by the iodine atom of the Cu­(I)-I chain, while the pyridine ring acts as an electron donor site, transferring the excited electron to CO_2_. However, the N 1s XPS signal of **TiO** **_2_** **@50%CP4** exhibits an additional shift compared to **CP4**, along with the emergence of a new contribution attributed to an N–C=O type environment (Table S3). This feature may correspond to an intermediate species formed during the CO_2_ reduction pathway, likely involving the amino (−NH_2_) group of the pyridine moiety rather than the coordinated imine group.

On the other hand, geometry optimizations reveal that, although the interaction is stronger, the O_2_C–I distances in **CP4** are larger than for **CP1–3**. This is not a problem for the electron transfer process since the excited electron is located at the organic part, as previously discussed. However, this larger distance in **CP4** may hinder the adsorption of the intermediates that should be stabilized to evolve to C_2_ products, such as ethanol, which could explain the higher selectivity of **TiO** **_2_** **@50%CP4** toward methanol compared to the other **TiO** **_2_** **@50%CPs**.

## 4 Conclusions

The studied one-dimensional Cu­(I)-I coordination polymers (CPs) with ladder-like chains stabilized by pyridine ligands exhibit high-energy VBE and CBE and band gaps of 2.87–3.54 eV. While these features do not enable efficient standalone CO_2_ photoreduction, their combination with commercially available TiO_2_ anatase nanoparticles produces highly active photocatalytic systems, yielding significantly higher alcohol production compared to benchmark materials. This performance is attributed to a Z-scheme mechanism, where photoexcited electrons in the CP conduction band and holes in the TiO_2_ valence band are spatially separated, maximizing the reductive and oxidative capacities. The optimized **TiO** **_2_** **@5%CP4** system achieves selective methanol production of 894 μg·g_cat_^–1^·h^–1^, surpassing **TiO** **_2_** **@3%CuO** (318 μg·g_cat_^–1^·h^–1^), while maintaining stability over 10 h. **CP4** offers enhanced chemical stability via amine-pyridine hydrogen bonding and a superior CO_2_ interaction, supported by adsorption experiments and DFT calculations.

Importantly, this system is constructed from inexpensive, abundant materials, avoiding noble metals or rare earths, and CPs are synthesized under mild, scalable conditions using water as a solvent. Future work will explore other Cu­(I)-I CPs with tunable electronic structures and porosities to further enhance photocatalytic efficiency, extend product selectivity, and enable continuous CO_2_ to alcohol conversion in large-scale reactors.

## Supplementary Material



## Notes

Detailed experimental procedures and computational details (PDF)
