# Supplementary material for: Enhancing CO2 to Alcohol Conversion: Powerful Photocatalysts Based on TiO2–Cu(I)-Iodine-Pyridine One-Dimensional Coordination Polymers
Source: Inorg Chem. 2025 Nov 11;64(46):22875–88. doi: 10.1021/acs.inorgchem.5c04083 (PMC12648666; doi:10.1021/acs.inorgchem.5c04083)
Supplement: Supplementary file 1 [file ic5c04083_si_001.pdf]

Supporting Information

Enhancing CO_2 to Alcohol Conversion: Powerful Photocatalysts Based on TiO_2-Cu(I)-Iodine-Pyridine 1D Coordination Polymers

Julian Avila-Duran[Δ], Jon Napal[α], Fernando Aguilar-Galindo[β, Ω] Oscar Castillo[α], Pilar Amo-Ochoa[Δ, Ω*]*

[Δ]*Inorganic Chemistry Department, Faculty of Sciences, Autonomous University of Madrid (UAM), 28049 Madrid, Spain.*

[α]*Department of Organic and Inorganic Chemistry, University of the Basque Country (UPV/EHU), P.O. 644, E-48080 Bilbao, Spain*

[β]*Chemistry Department, Faculty of sciences, Autonomous University of Madrid (UAM), 28049 Madrid, Spain.*

[Ω]*Institute for Advanced Research in Chemical Sciences (IAdChem). Autonomous University of Madrid (UAM), 28049 Madrid, Spain*

E-mail: pilar.amo@uam.es / oscar.castillo@ehu.eus

Experimental details

SCXRD of CPs

Table S1. Crystallographic parameters of compounds **CP4** and **CP5**.

	**CP4**	**CP5**
**Formula**	$C_6H_8CuI N_2$	$C_6H_6ClCuIN$
**D_{calc}/ g.cm^{-3}**	2.374	2.489
**μ/ mm^{-1}**	6.236	6.448
**Formula Weight**	298.58	318.01
**Colour**	Clear colourless	Clear colourless
**Shape**	Needle	Needle
**Size/ mm^3**	0.18x0.11x0.05	0.252x0.058x0.038
**T/ K**	295	295
**Crystal system**	Monoclinic	Triclinic
**Space group**	P 2_1/n	P -1
***a*/ Å**	11.4046(3)	4.30550(10)
***b*/ Å**	4.2166(1)	10.0703(2)
***c*/ Å**	17.9223(5)	11.0726(2)
**α/ º**	90	113.967(2)
**β/ º**	104.244(3)	93.021(2)
**γ/ º**	90	101.841(2)
**V/ Å^3**	835.36(4)	424.357(17)
**Z**	4	2
**Measured Ref.**	15051	15591
**Independent Refl.**	1521	1554
**GooF**	0.961	1.063
**wR^2 (all data)**	0.0421	0.0518
**wR^2**	0.0415	0.0510
**R_1 (all data)**	0.0175	0.0208
**R_1**	0.0166	0.0196

Table S2. More relevant structural parameters of compound **CP1-5**.

	**CP1**	**CP2**	**CP3**	**CP4**	**CP5**
**Cu-N (Å)**	2.047	2.063	2.051	2.039	2.073
**Cu-I_{step} (Å)**	2.640	2.661	2.638	2.656	2.673
**Cu-I_{upper} (Å)**	2.689	2.694	2.693	2.745	2.742
**Cu-I_{lower} (Å)**	2.644	2.651	2.653	2.661	2.648
**Cu···Cu (Å)**	2.874	2.911	2.855	2.778, 3.401	2.696, 3.517
**I···I (Å)**	4.461	4.469	4.479	4.196, 4.534	4.119, 4.588
**Cu-I-Cu (°)**	65.9, 65.3	66.5, 66.8	65.3, 64.7	63.0, 78.0	60.9, 81.0
**I-Cu-I (°)**	113.7-115.2	113.1-114.6	114.3-115.7	102.0, 117.0	99.0, 119.1
**Dihedral Cu_2I_2 (°)**	119.9	120.8	119.9	110.9	114.1
**Torsion I_{step}-Cu-N-C (°)**	32.7	31.5	32.9	44.9	49.3

FTIR-ATR of CPs

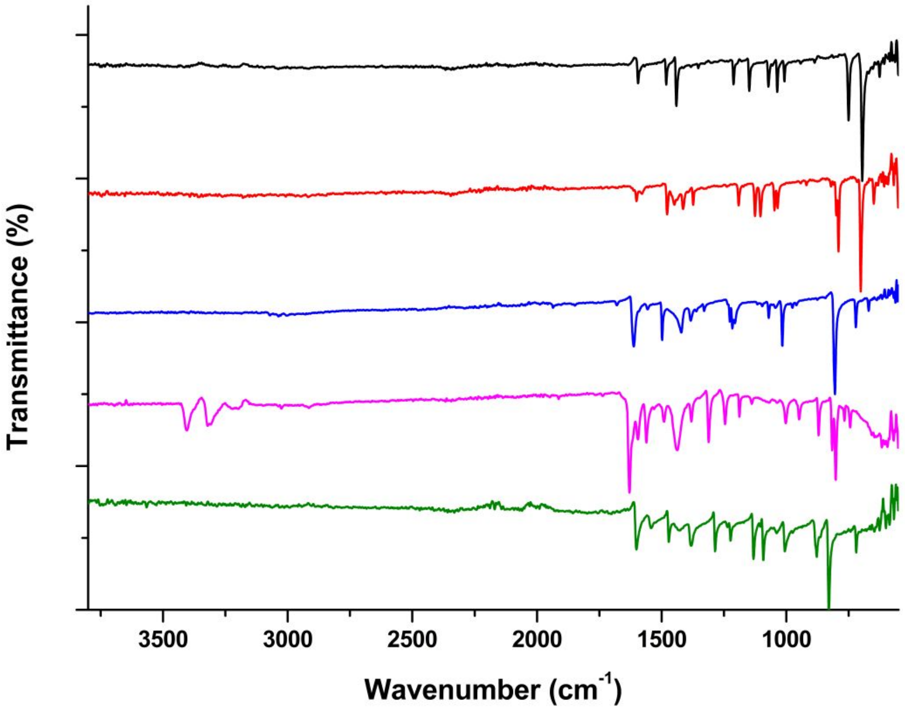

Figure S1. FTIR-ATR spectrum of microcrystalline CP powders: **CP1** (black line), **CP2** (red line), **CP3** (blue line), **CP4** (pink line) and **CP5** (green line).

PXRD of CPs

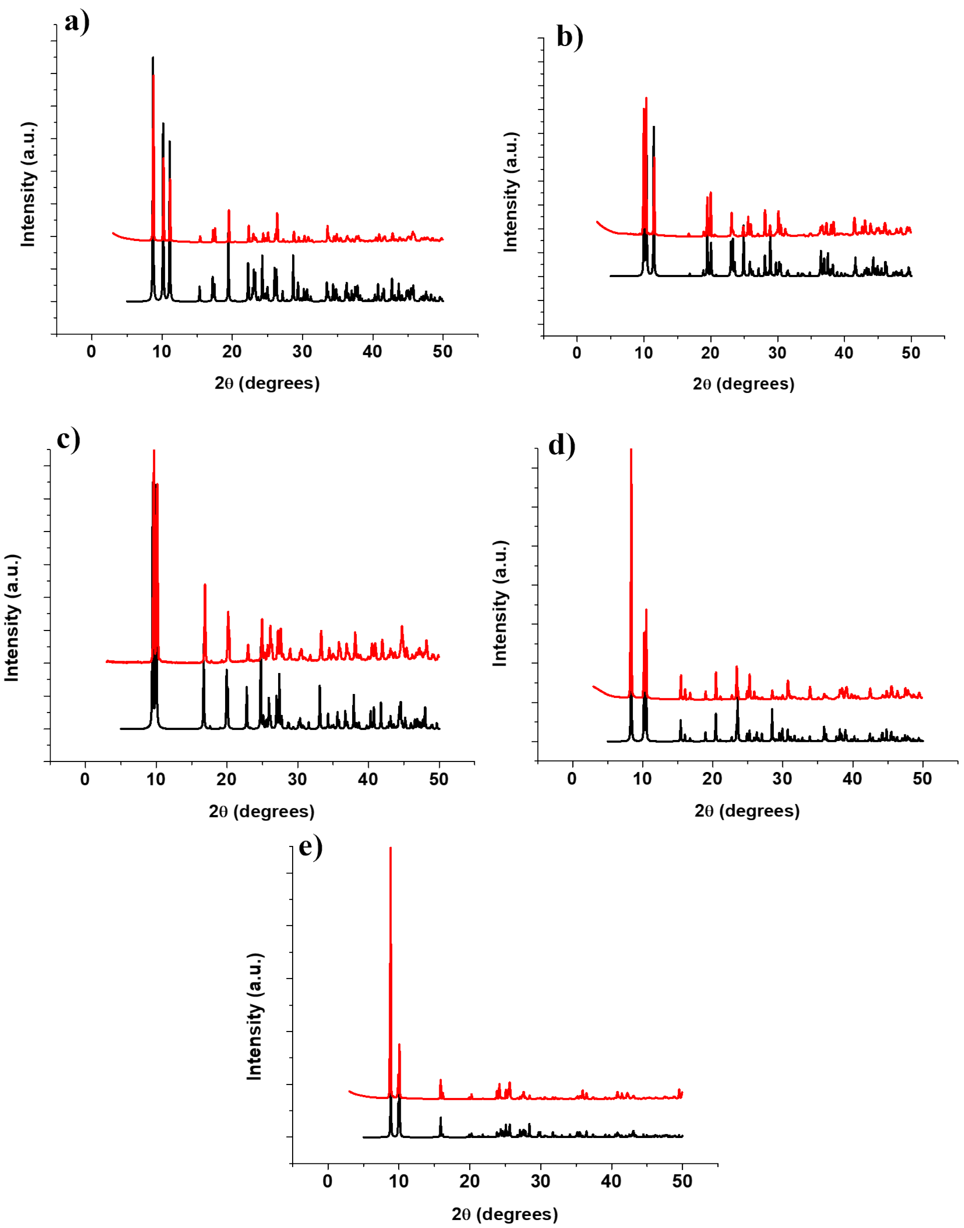

Figure S2. PXRD of theoretical (black line) and experimental (red line) of a) **CP1**, b) **CP2**, c) **CP3**, d) **CP4** and e) **CP5**.

Morphology characterization of CPs

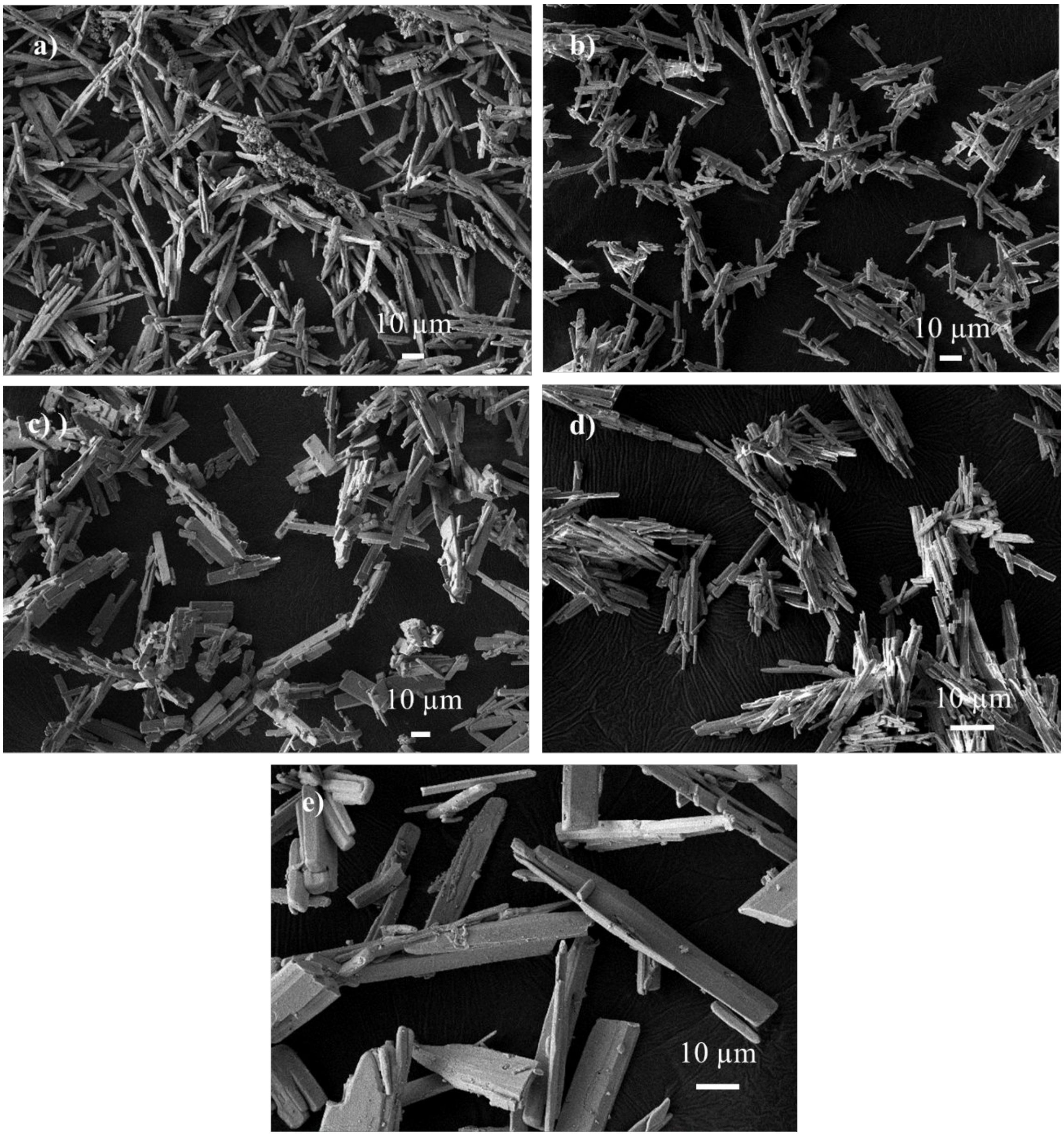

Figure S3. SEM micrographs of a) **CP1**, b) **CP2**, c) **CP3**, d) **CP4** and e) **CP5** microcrystals.

Thermal characterization of CPs

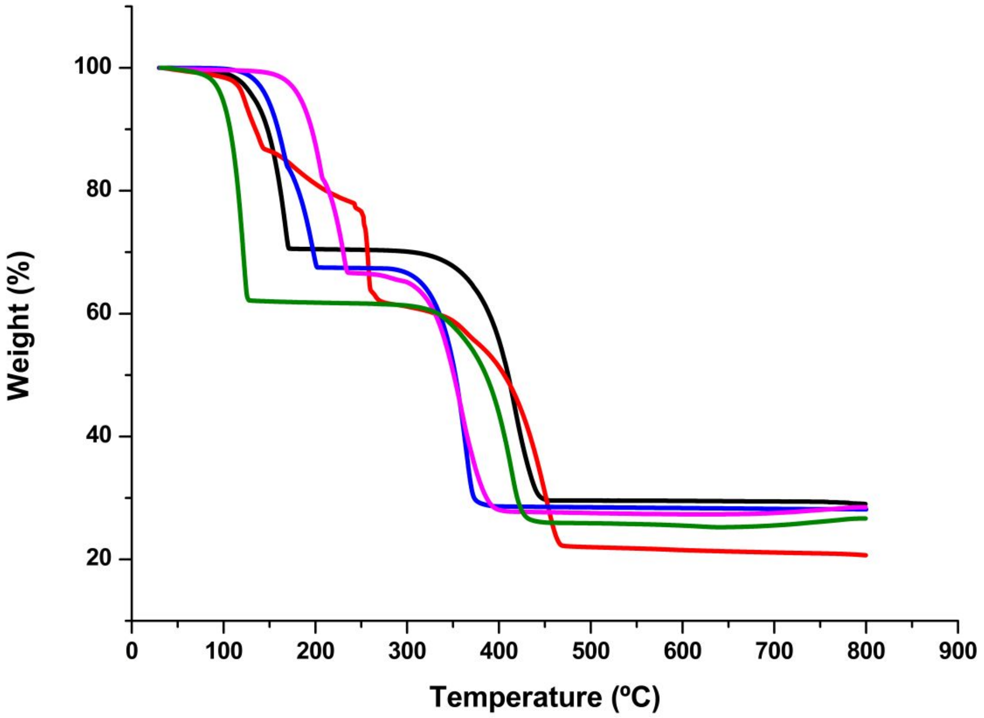

Figure S4. TGA analysis of **CP1** (black line), **CP2** (red line), **CP3** (blue line), **CP4** (pink line) and **CP5** (green line), under synthetic air (80% N_2, 20% O_2).

Morphology characterization of ground CPs

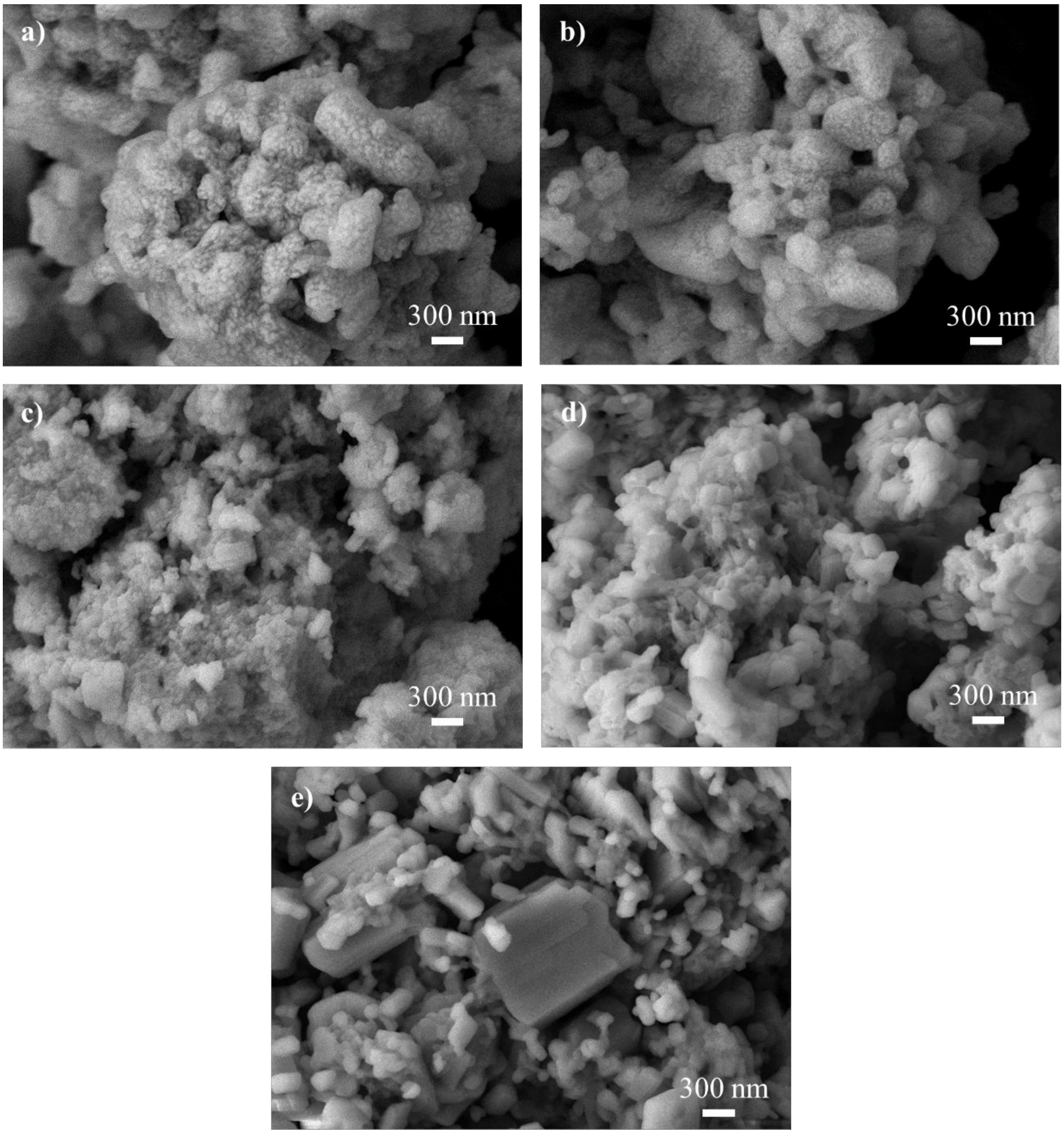

Figure S5. SEM micrographs of a) **CP1**, b) **CP2**, c) **CP3**, d) **CP4** and e) **CP5** obtained after grinding for 5 min.

FTIR-ATR of ground CPs

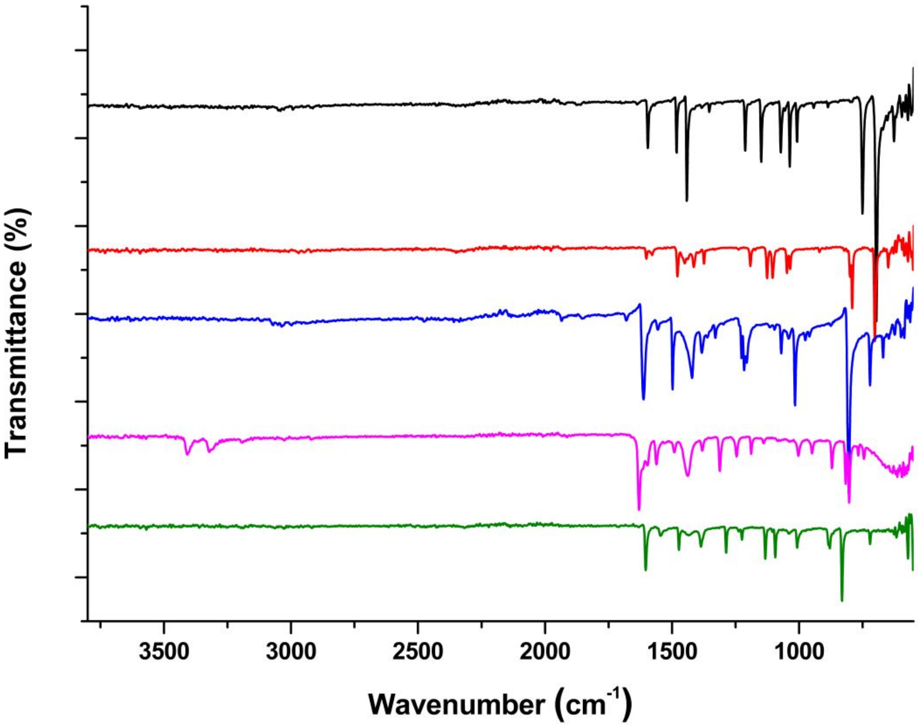

Figure S6. FTIR-ATR spectrum of ground CP: **CP1** (black line), **CP2** (red line), **CP3** (blue line), **CP4** (pink line) and **CP5** (green line).

PXRD of ground CPs

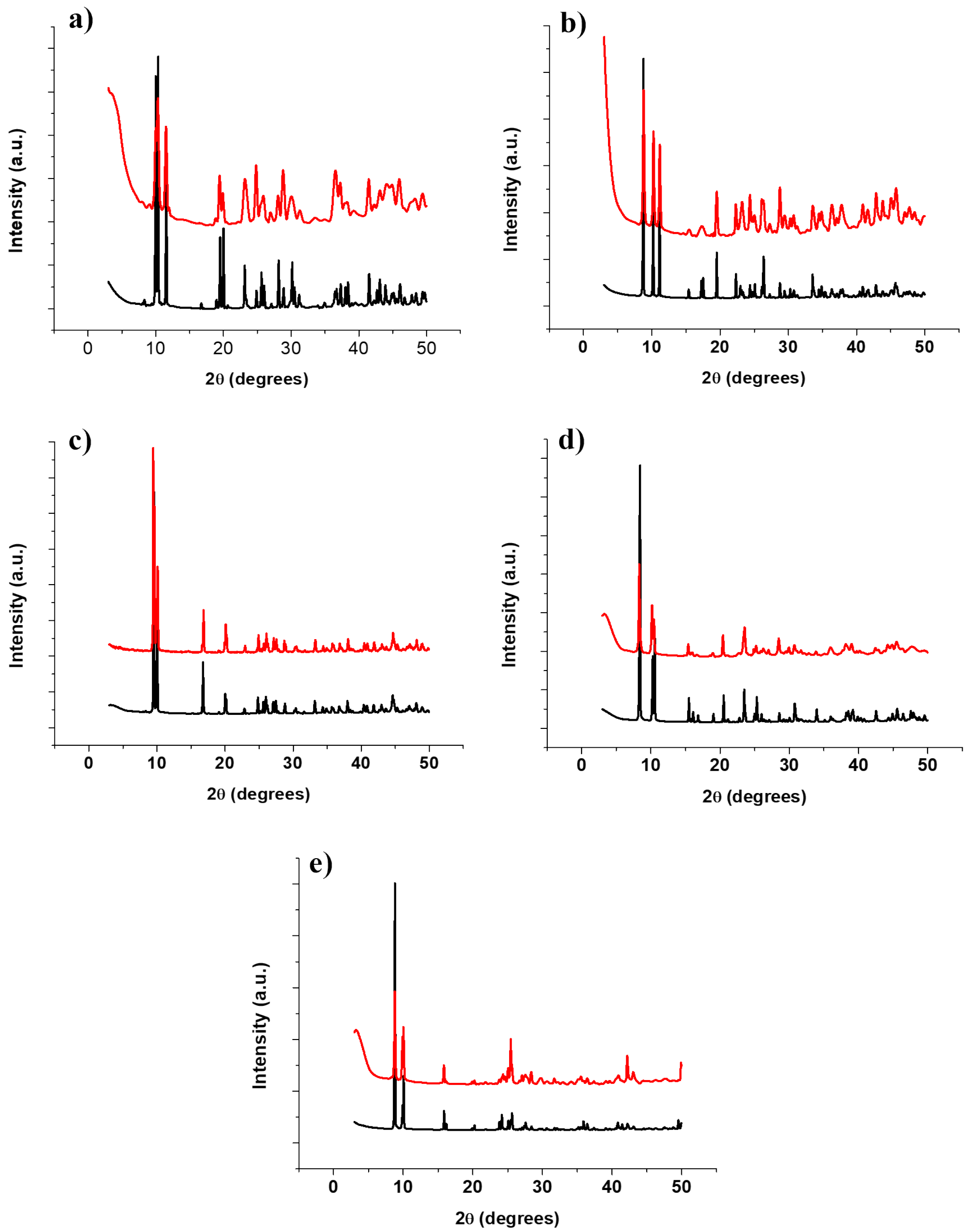

Figure S7. Experimental PXRD of microcrystalline CP (black line) and ground CP (red line) of a) **CP1**, b) CP2, c) **CP3**, d) **CP4** and e) **CP5**.

Morphology of TiO_2 nanoparticles

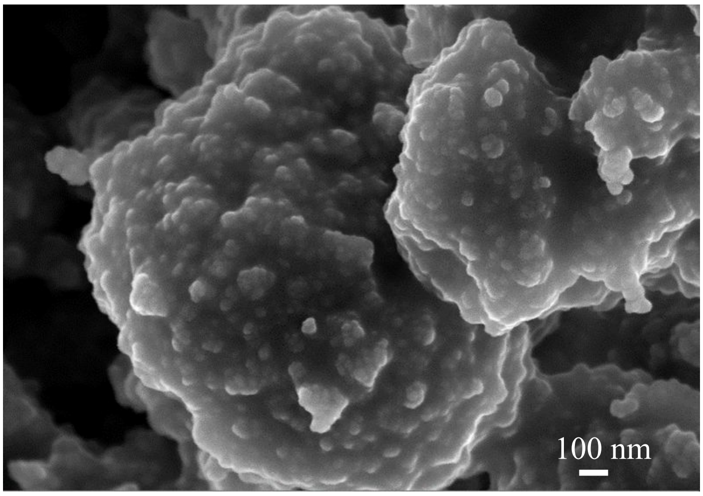

Figure S8. SEM micrographs of TiO_2 nanoparticles.

Morphology of ground TiO_2@50%CP

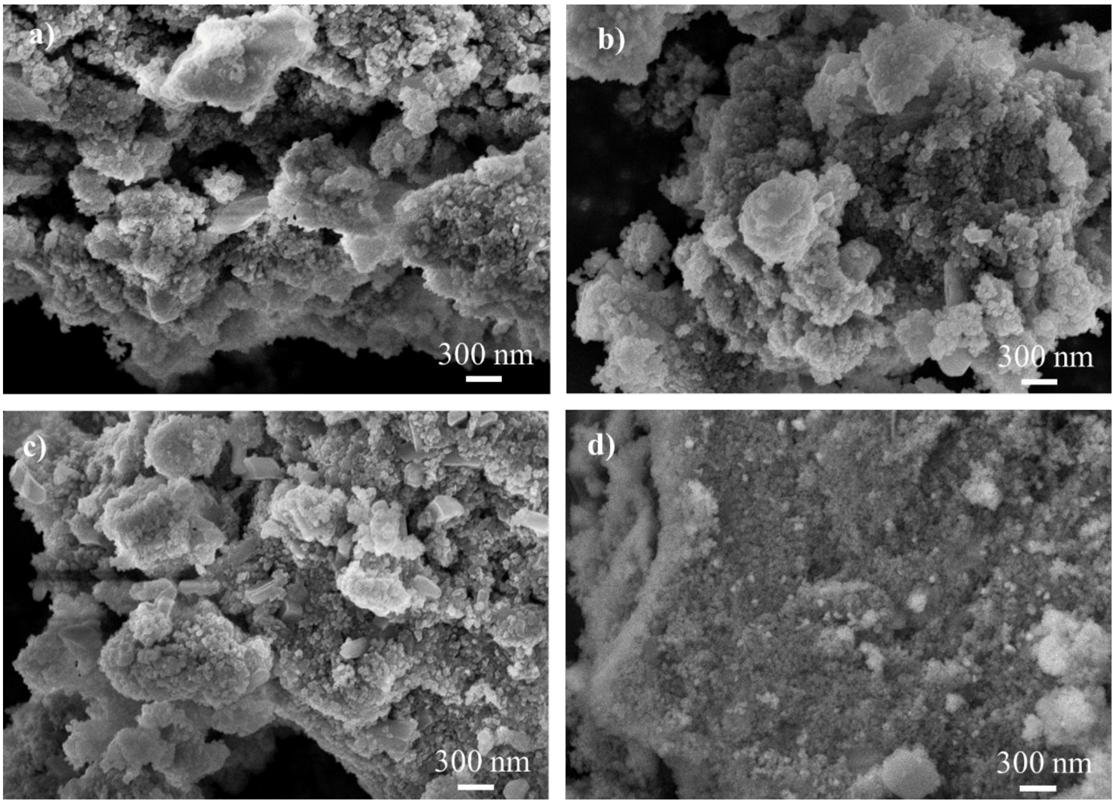

Figure S9. SEM micrographs of **TiO_2@50%CP** systems with a) **CP1**, b) **CP2**, c) **CP3** and d) **CP4** obtained after grinding for 5 min.

Elemental distribution of ground TiO_2@50%CP

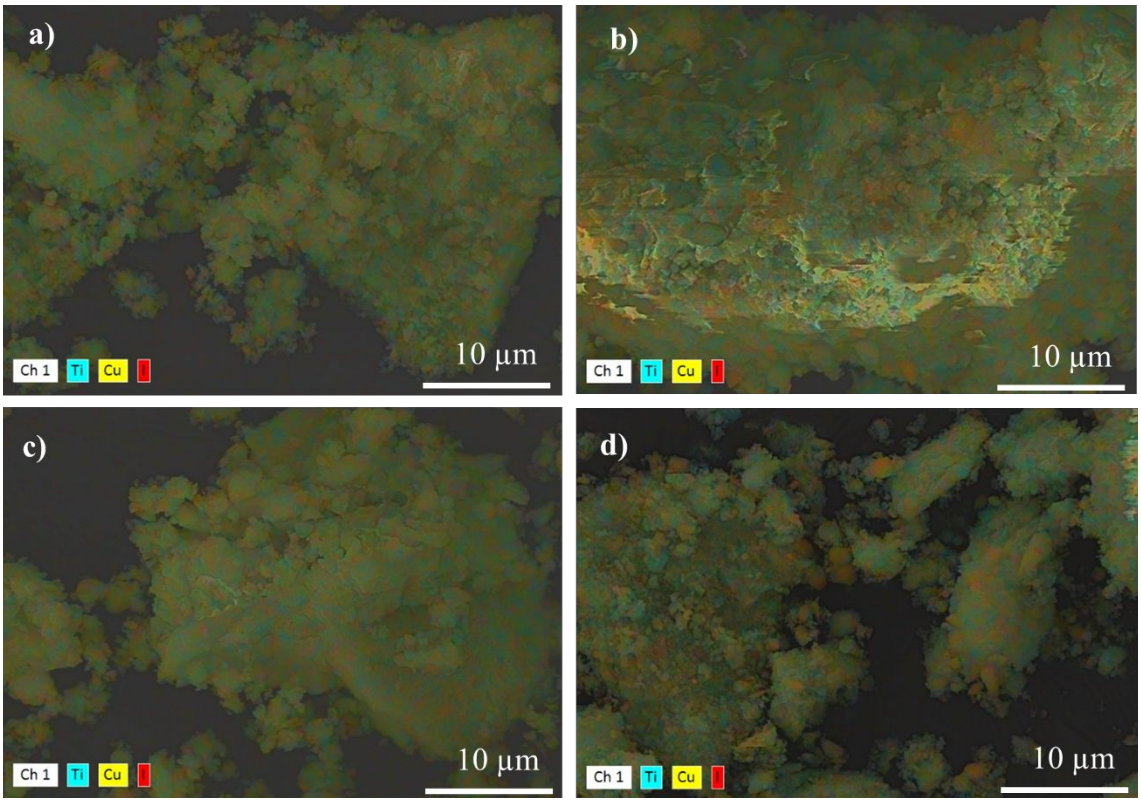

Figure S10. Elemental distribution by SEM-EDX of **TiO_2@50%CP** systems with a) **CP1**, b) **CP2**, c) **CP3** and d) **CP4** obtained after grinding for 5 min.

FTIR-ATR of ground TiO_2@50%CP

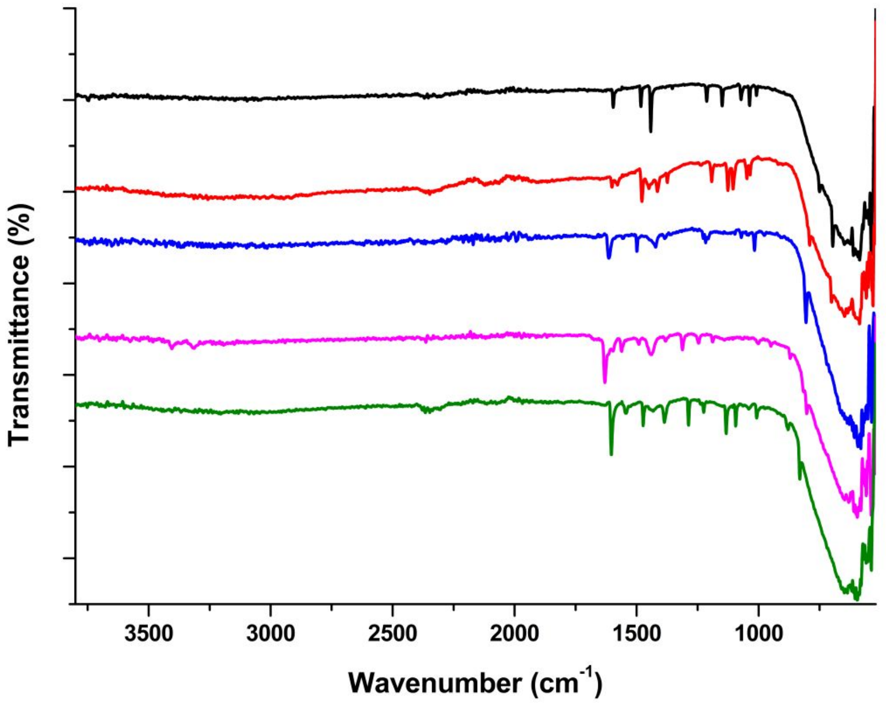

Figure S11. FTIR-ATR spectra of **TiO_2@50%CP** systems with each CP: **CP1** (black line), **CP2** (red line), **CP3** (blue line), **CP4** (pink line) and **CP5** (green line).

PXRD of ground TiO_2@50%CP

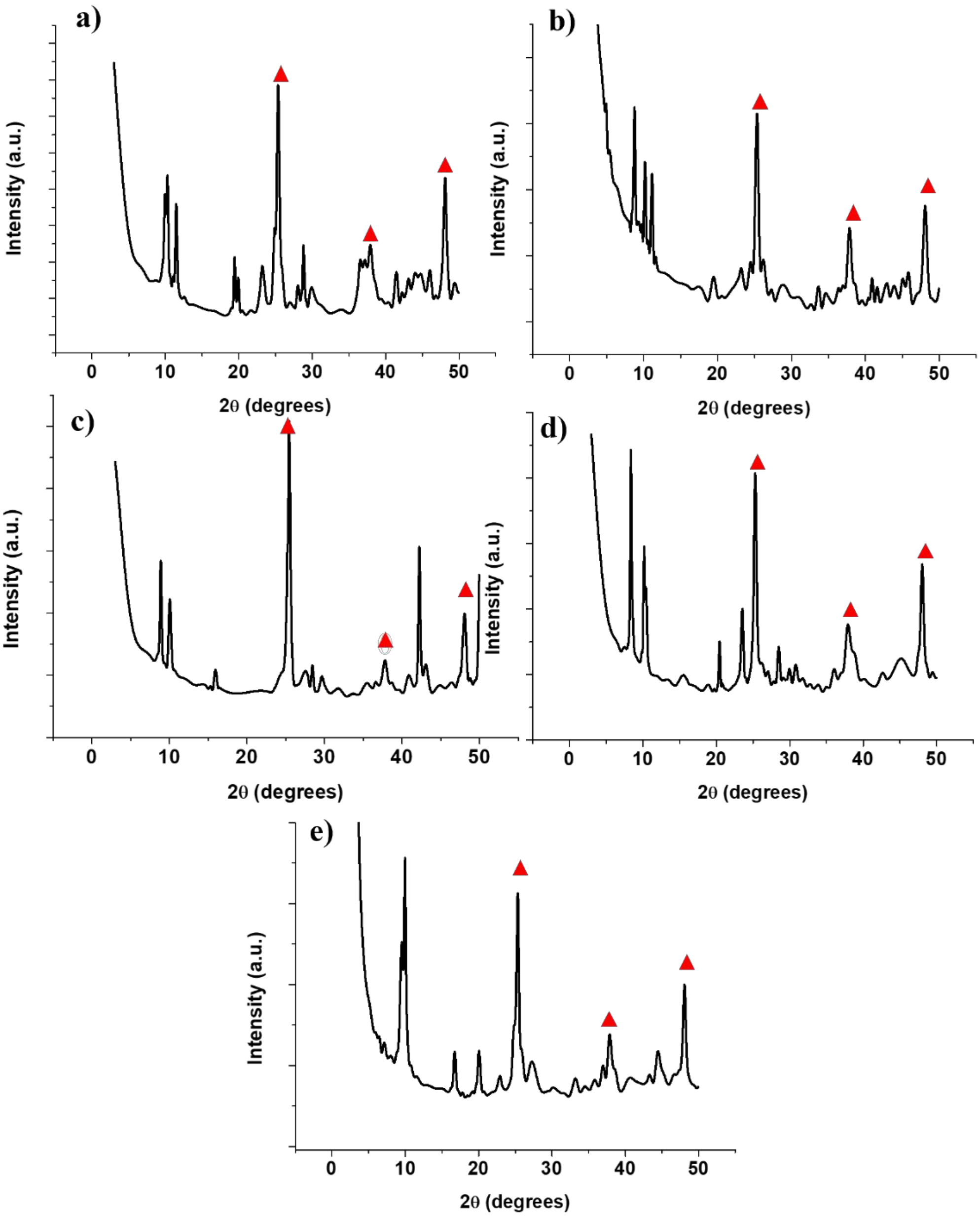

Figure S12. PXRD of experimental **TiO_2@50%CP** systems with each CP of a) **CP1**, b) **CP2**, c) **CP3**, d) **CP4** and e) **CP5**. The red triangles are the peaks corresponding to the TiO_2.

Optical characterization

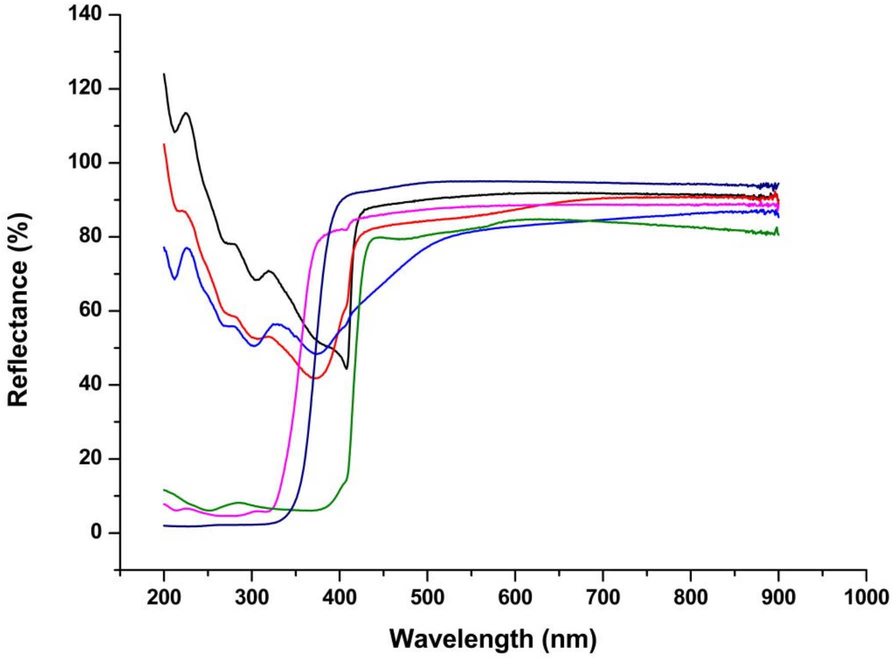

Figure S13. Experimental diffuse reflectance spectra of the TiO_2 and microcrystalline CP: **CP1** (black), **CP2** (red), **CP3** (blue), **CP4** (pink), **CP5** (green) and $\mathbf{TiO_2}$ (brown).

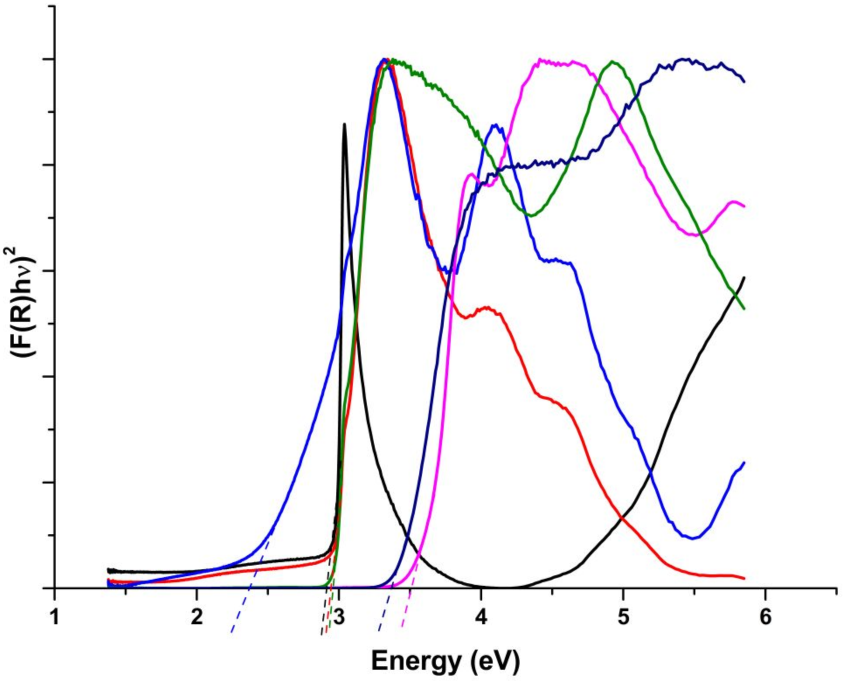

Figure S14. Tauc plots obtained with the Kubelka-Munk function and the linear fit for optical band gaps of the TiO_2 and microcrystalline CP: **CP1** (black), **CP2** (red), **CP3** (blue), **CP4** (pink) and **CP5** (green) and $\mathbf{TiO_2}$ (brown).

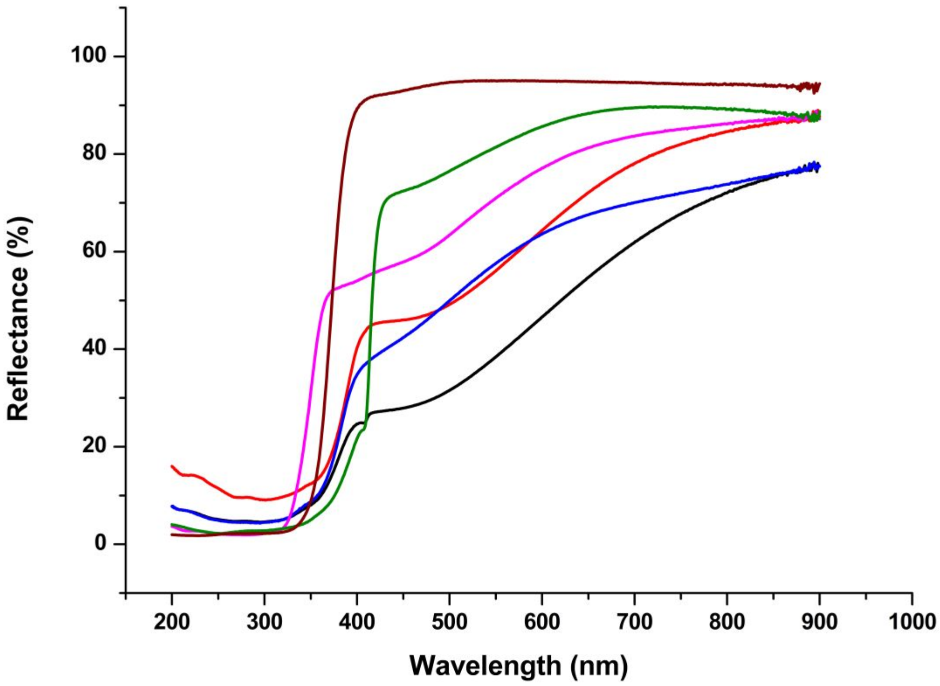

Figure S15. Experimental diffuse reflectance spectra of the ground TiO_2 and ground CP: **CP1** (black), **CP2** (red), **CP3** (blue), **CP4** (pink) and **CP5** (green) and **TiO_2** (brown).

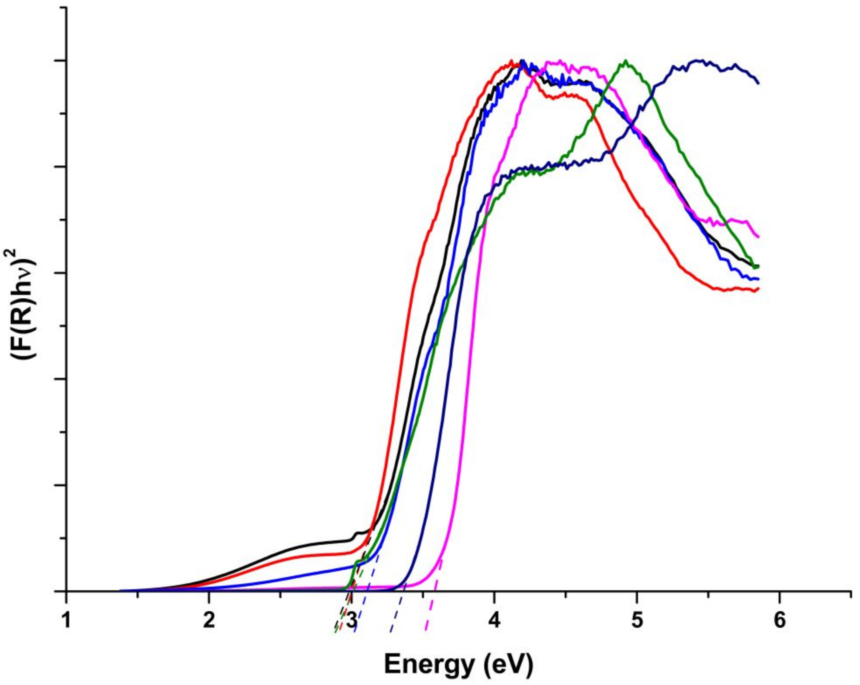

Figure S16. Tauc plots obtained with the Kubelka-Munk function and the linear fit for optical band gaps of the ground TiO_2 and ground polymer: **CP1** (black), **CP2** (red), **CP3** (blue), **CP4** (pink) and **CP5** (green) and **TiO_2** (brown).

FTIR-ATR of ground TiO_2@50%CP after CO_2 photoreduction

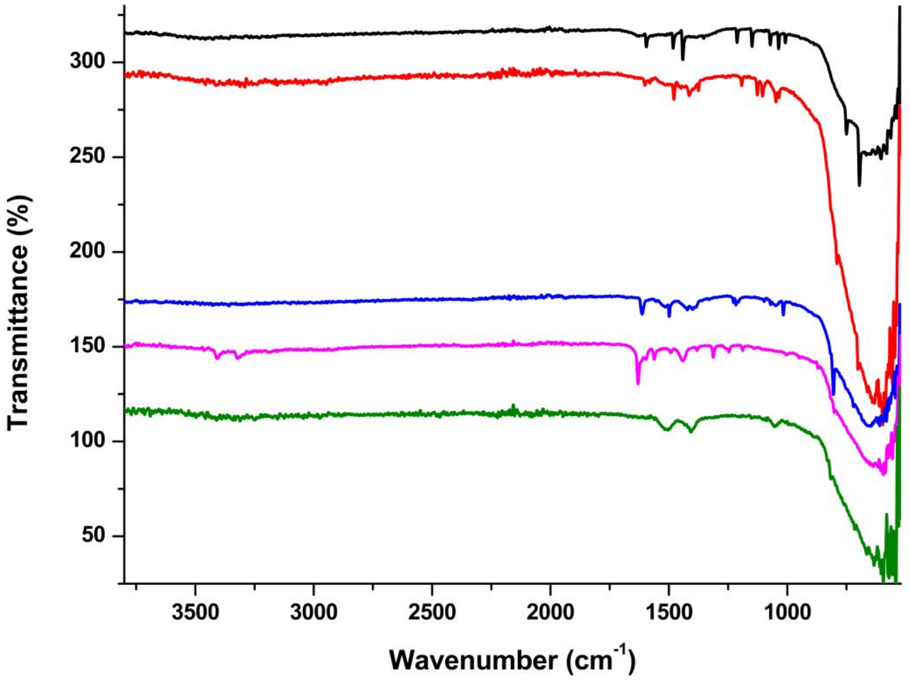

Figure S17. FTIR-ATR spectra of the **TiO_2@50%CP** systems after 10h of CO_2 photoreduction with each CP: **CP1** (black line), **CP2** (red line), **CP3** (blue line), **CP4** (pink line) and **CP5** (green line).

PXRD of ground TiO_2@50%CP after CO_2 photoreduction

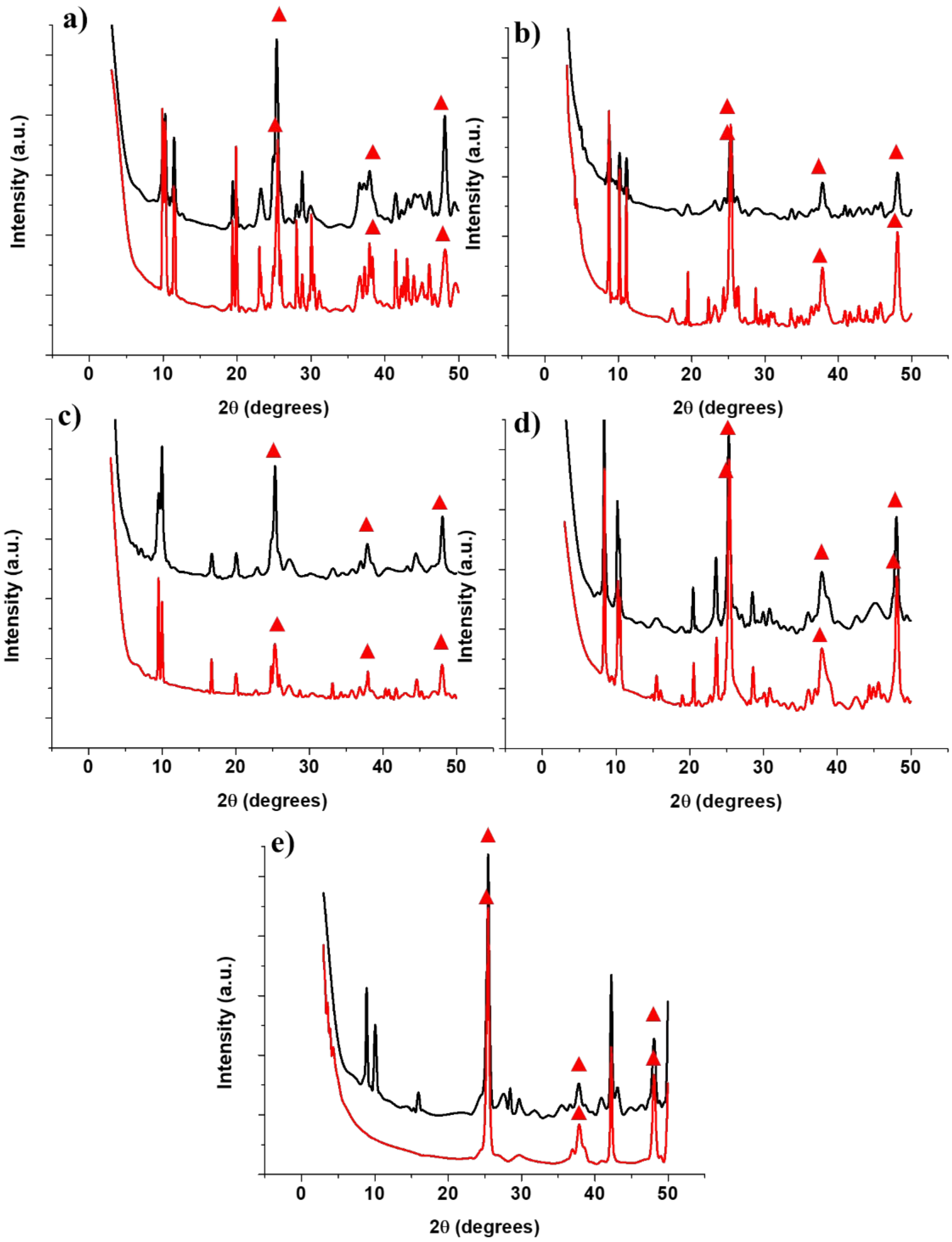

Figure S18. PXRD of the experimental **TiO_2@50%CP** systems with each polymer (black line) and after 10 h of CO_2 photoreduction (red line) of a) **CP1**, b) **CP2**, c) **CP3**, d) **CP4** and e) **CP5**. The red triangles are the peaks corresponding to TiO_2.

Morphology of ground TiO_2@50%CP after CO_2 photoreduction

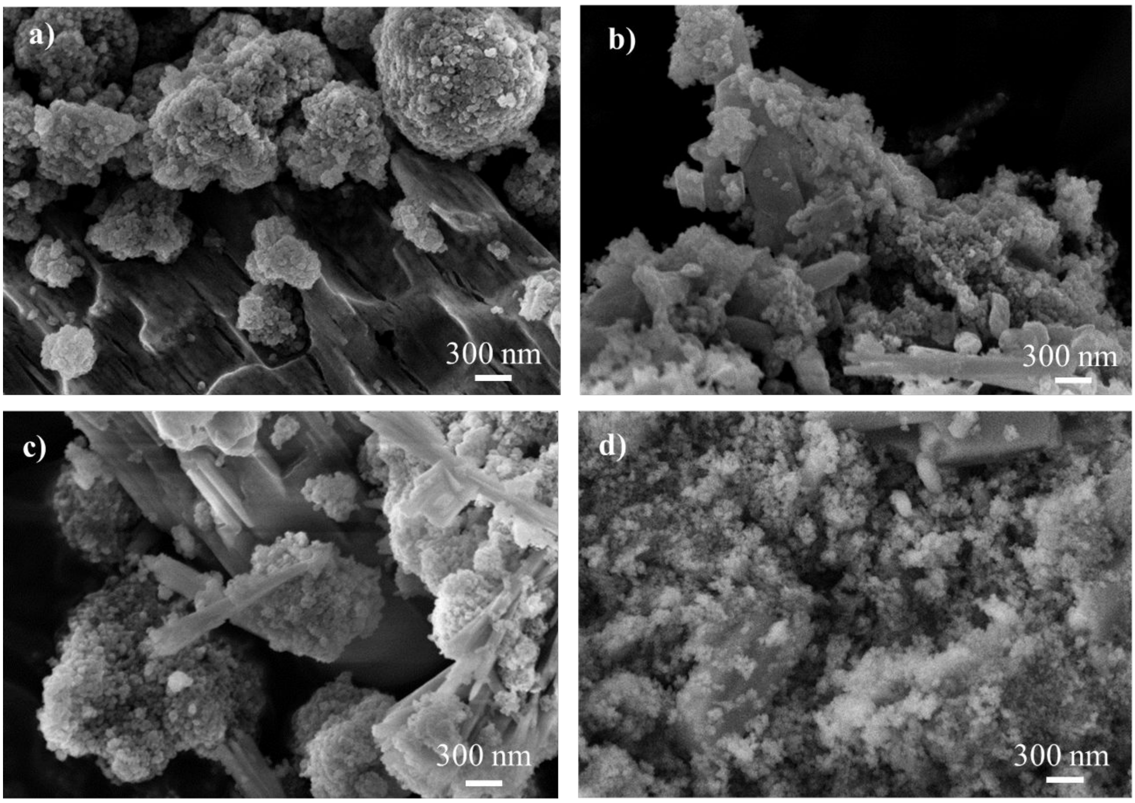

Figure S19. SEM micrographs of **TiO_2@50%CP** systems with a) **CP1**, b) **CP2**, c) **CP3** and d) **CP4** after 10 h of CO_2 photoreduction.

Elemental distribution of ground TiO_2@50%CP after CO_2 photoreduction

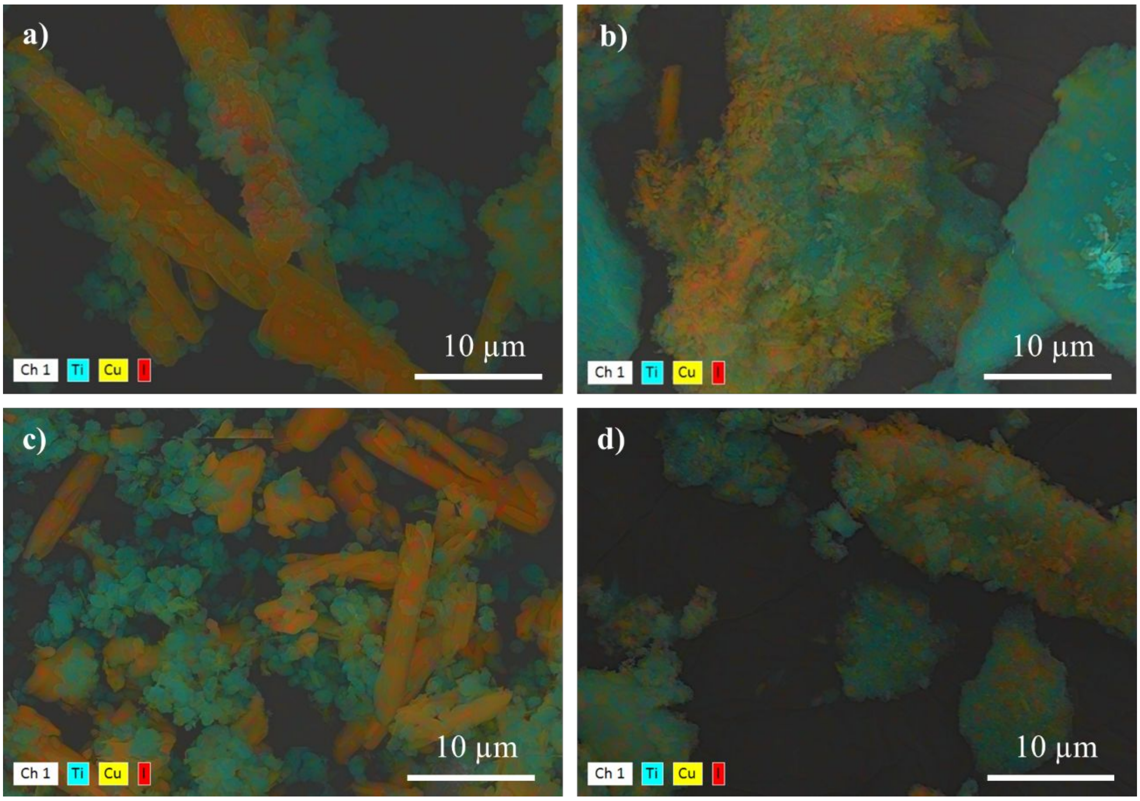

Figure S20. Elemental distribution by SEM-EDX of **TiO_2@50%CP** systems with a) **CP1**, b) **CP2**, c) **CP3** and d) **CP4** after 10 h of CO_2 photoreduction.

Surface area and CO_2 adsorption

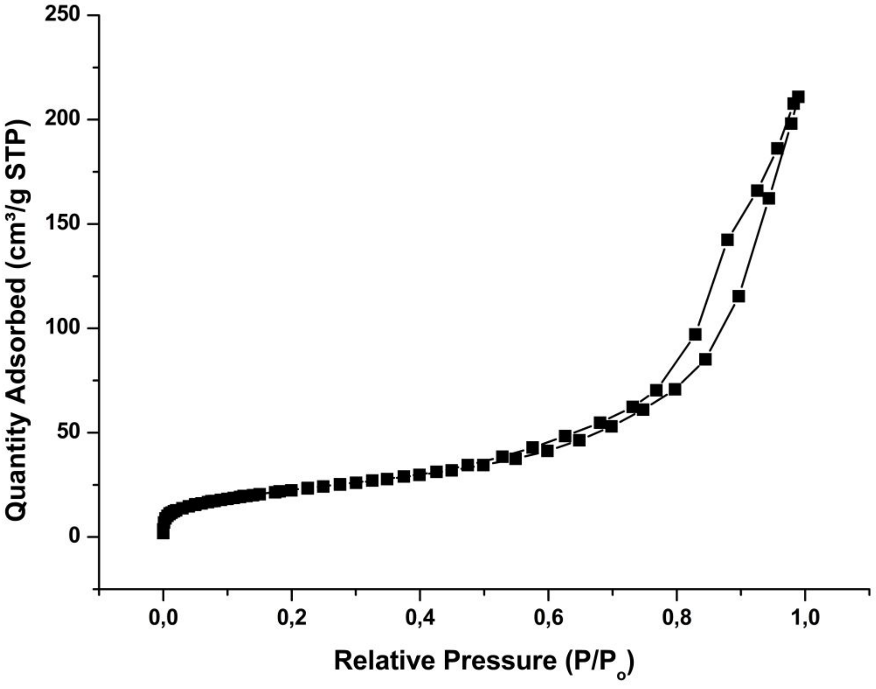

Figure S21. N_2 adsorption-desorption isotherms for TiO_2.

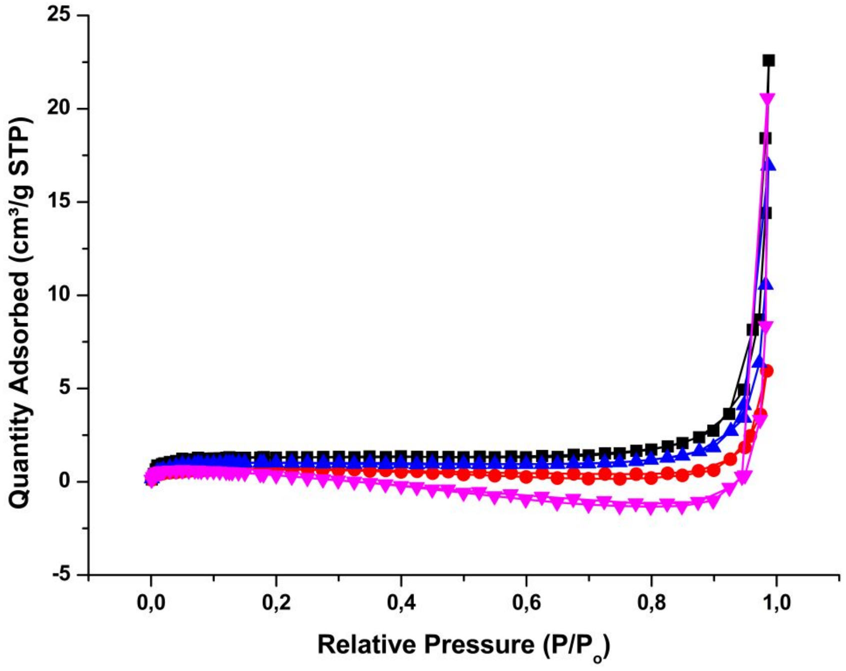

Figure S22. N_2 adsorption-desorption isotherms for ground CP: **CP1** (black line), **CP2** (red line), **CP3** (blue line) and **CP4** (pink line).

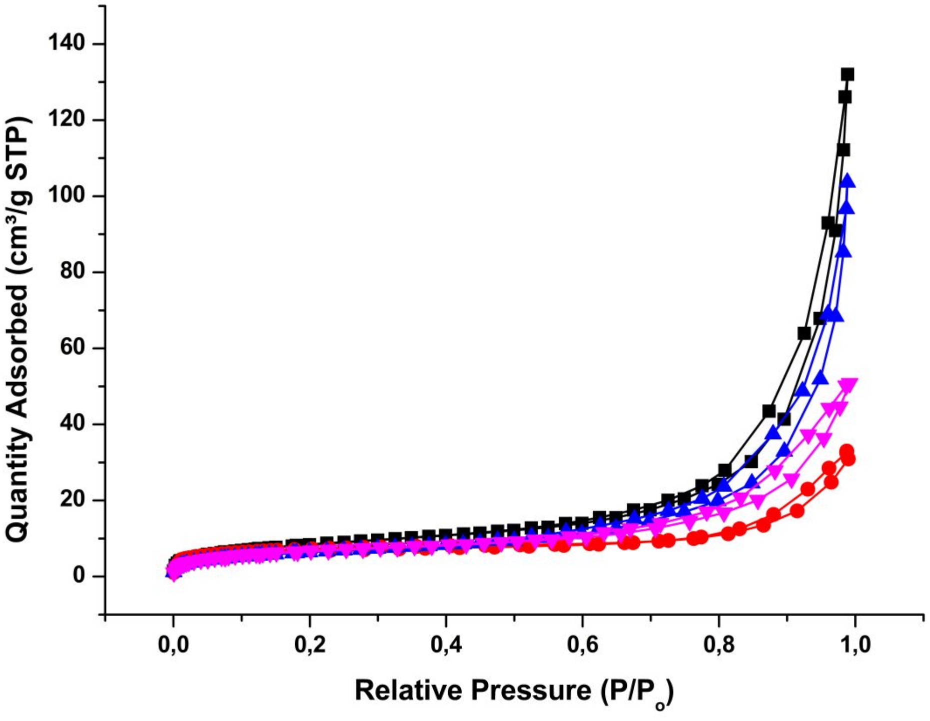

Figure S23. N_2 adsorption-desorption isotherms of **TiO_2@50%CP**: **CP1** (black line), **CP2** (red line), **CP3** (blue line) and **CP4** (pink line).

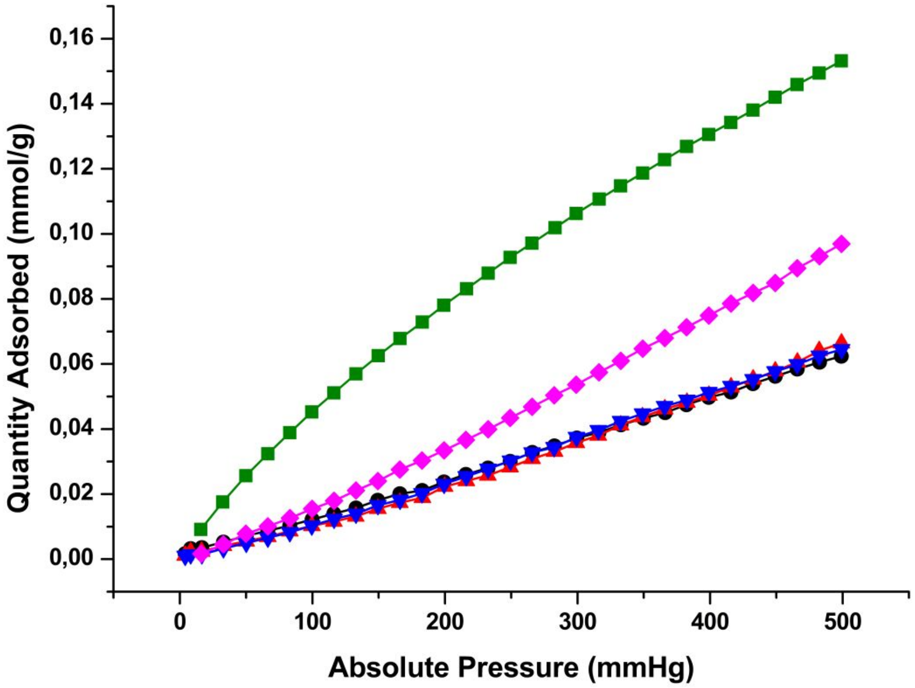

Figure S24. CO_2 adsorption isotherms of TiO_2 (green line) and **TiO_2@50%CP**: **CP1** (black line), **CP2** (red line), **CP3** (blue line) and **CP4** (pink line).

Surface characterization

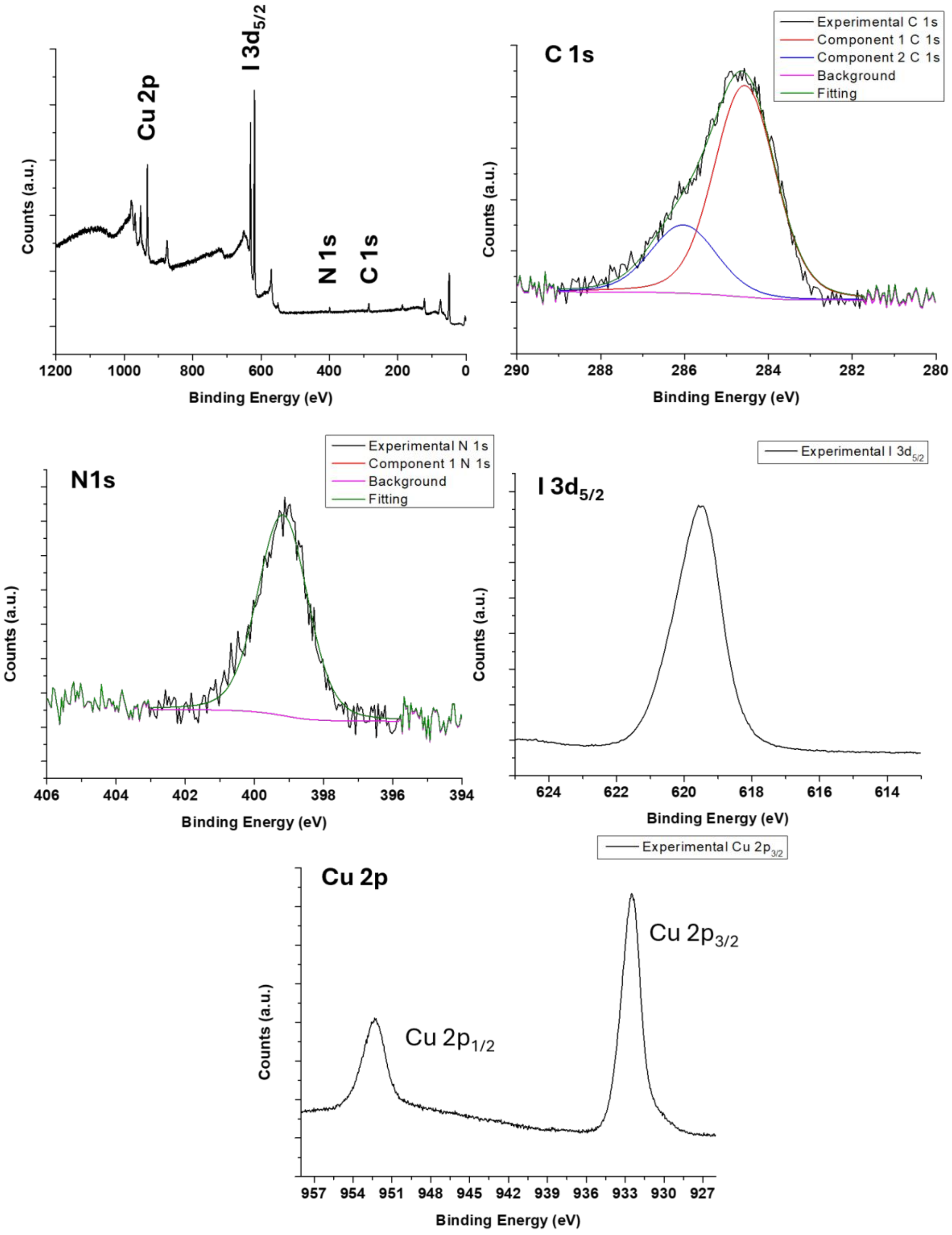

Figure S25. XPS survey spectrum of CP4.

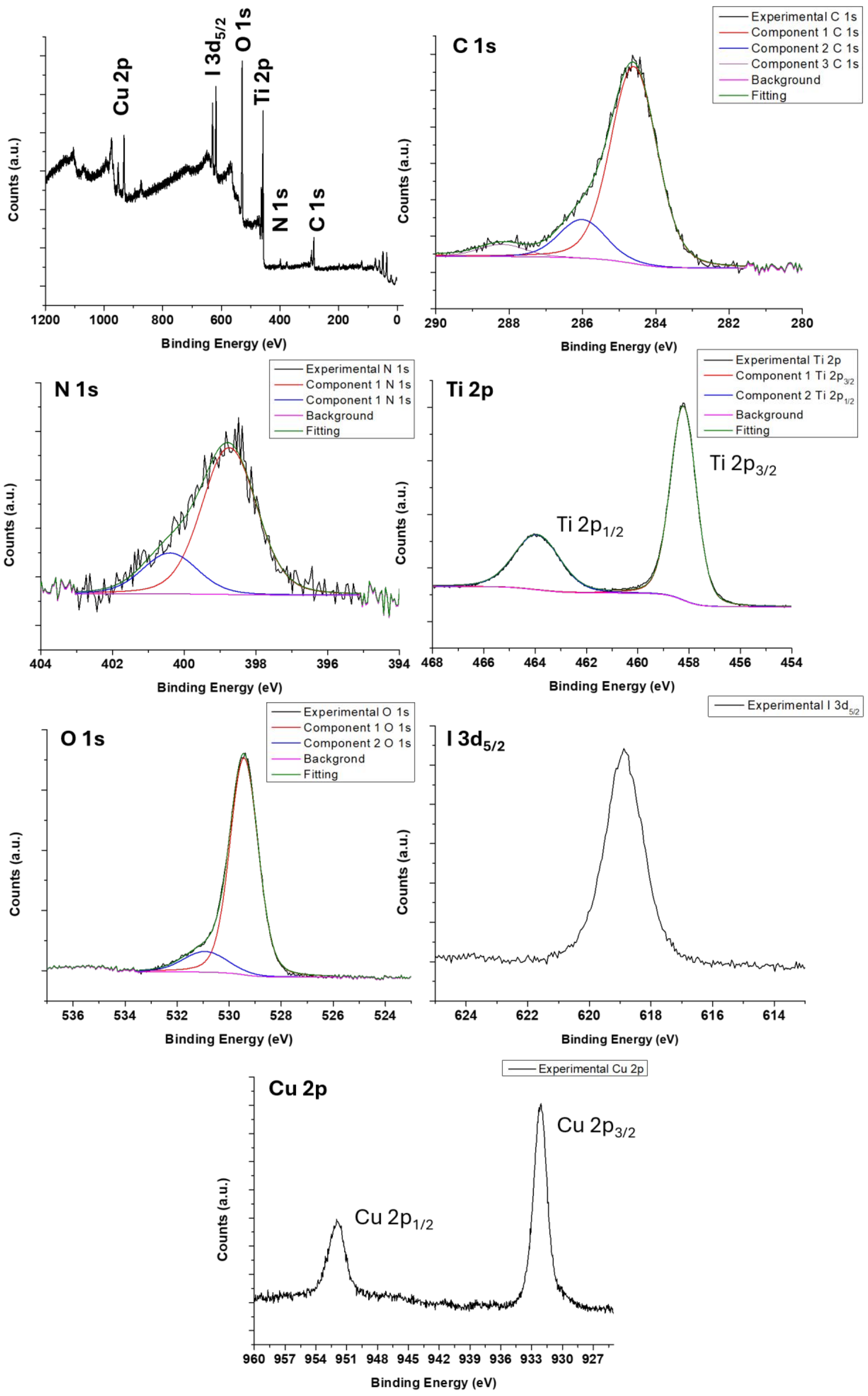

Figure S26. XPS survey spectrum of **TiO_2@50%CP4** after photocatalytic reduction.

Table S3. Assignment of the peaks obtained in XPS.

**CP4**			**TiO_2@50%CP4**		
**Element**	**Assignment**	**Position (eV)**	**Element**	**Assignment**	**Position (eV)**
**C**	C 1s (C-C, C-H)	284.6	**C**	C 1s (C-C, C-H)	284.6
	C 1s (C-N)	286.0		C 1s (C-N)	286.0
	-	-		C 1s (C=O)	288.2
**Cu**	Cu $2p_{3/2}$ (I)	932.5	**Cu**	Cu $2p_{3/2}$ (I)	932.2
	Cu $2p_{1/2}$ (I)	952.3		Cu $2p_{1/2}$ (I)	951.9
**I**	I $3d_{5/2}$	619.5	**I**	I $3d_{5/2}$	618.9
**N**	N 1s (pyridine)	399.2	**N**	N 1s (pyridine)	398.8
	-	-		N 1s (N-C=O)	400.4
			**Ti**	Ti $2p_{3/2}$ (IV)	458.2
				Ti $2p_{1/2}$ (IV)	463.9
			**O**	O 1s (Ti-O)	529.4
				O 1s (O-C=O)	530.9

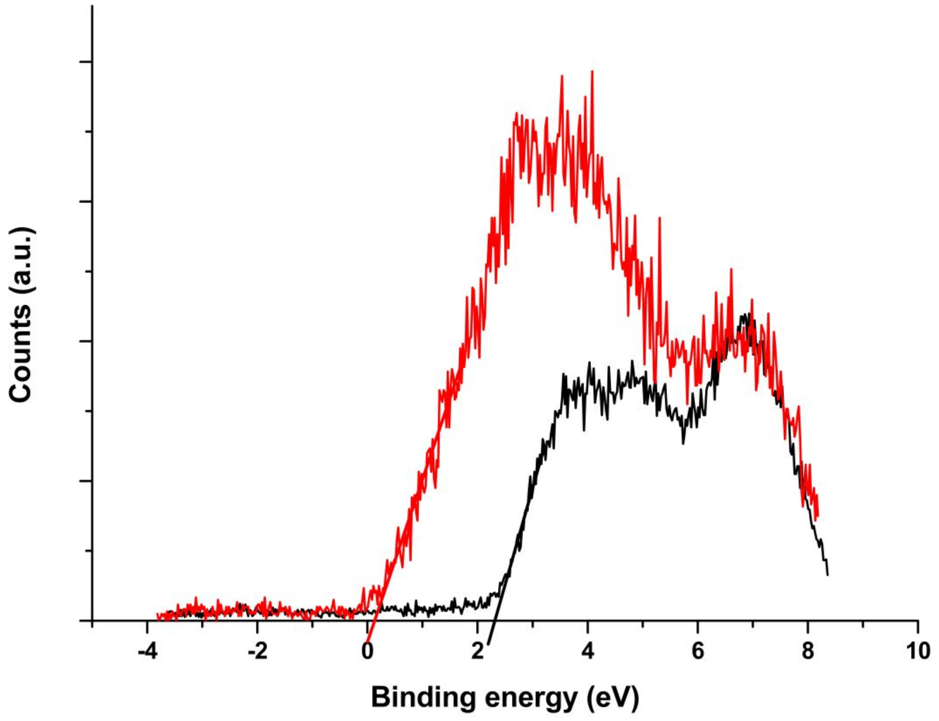

Figure S27. Representation of XPS low binding energy region of TiO_2 (black line) and **TiO_2@50%CP4** (red line). Energy values referent to the Fermi level.

Density Functional Theory (DFT) calculations

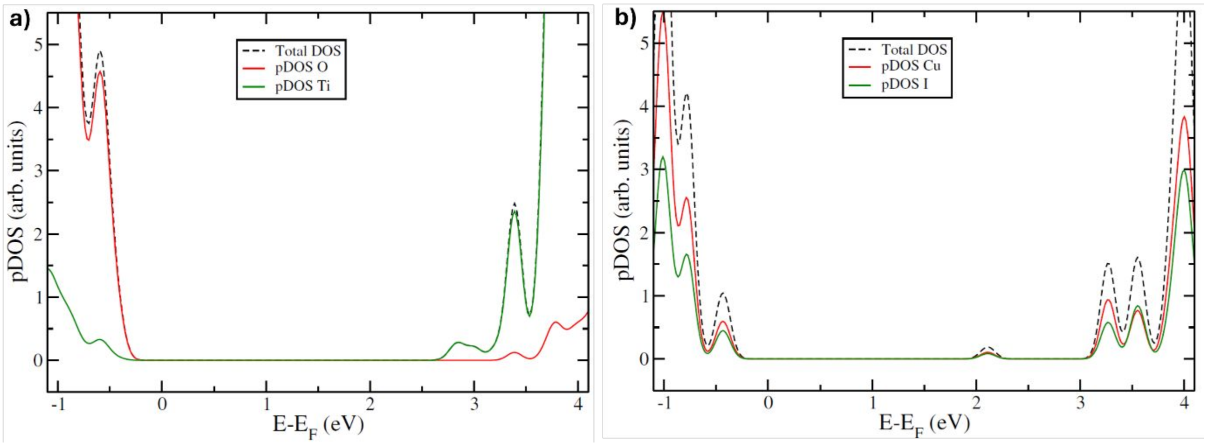

Figure S28. Density of states of: a) TiO_2 with projection on Ti and O unit atoms and b) CuI with projection on the Cu and I unit atoms.

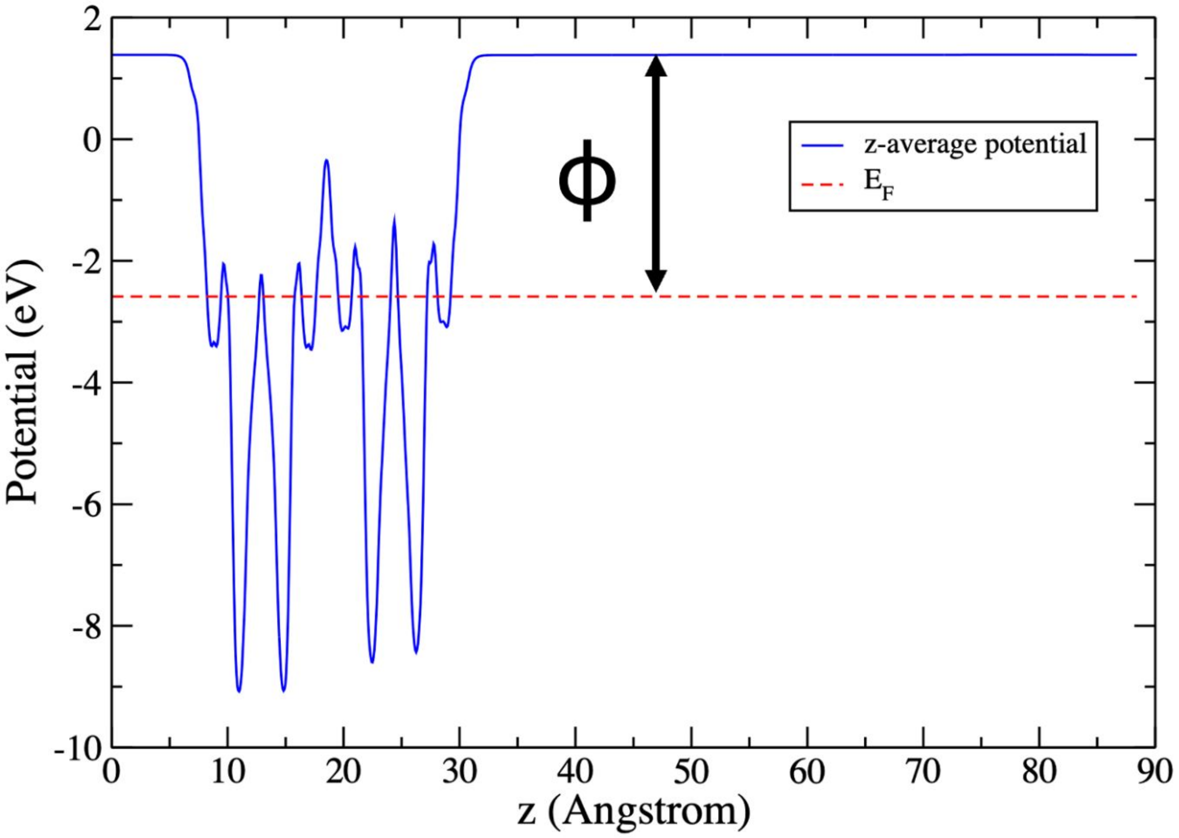

Figure S29. Z-averaged Hartree potential as for **CP4** surface. Dashed line indicates Fermi level. Work function is calculated as difference of the asymptotic potential and the Fermi level.

Table S4. Computationally calculated values of work function, valence band edge (referred to the vacuum and NHE), theoretical band gap and conduction band edge (NHE).

	Work function (eV)	Valence band (eV)	Valence band (V) vs NHE	Theoretical band gap (eV)	Conduction band (V) vs NHE
**TiO_2**	7.820	-8.22	3.72	3.20	0.52
**CuI**	6.740	-7.04	2,54	3.60	-1.06
**CP1**	3.778	-4.18	-0.32	2.80	-3.12
**CP2**	3.638	-4.04	-0.46	2.80	-3.26
**CP3**	3.597	-4.00	-0.50	2.70	-3.20
**CP4**	3.972	-4.37	-0.13	3.40	-3.53

Table S5. Experimental values obtained from the literature of the valence band edge (referred to the vacuum and NHE), band gap and conduction band edge (NHE). Data source for **TiO_2@50%CP4**: low binding energy XPS and DRS.

	Valence band (eV)	Valence band (V) vs NHE	Experimental band gap (eV)	Conduction band (V) vs NHE
**TiO_2** [88]	-7.41	2.91	3.20	-0.29
**Cu_2O** [88]	-6.42	1.92	2.20	-0.28
**CuO** [88]	-6.66	2.16	1.70	0.46
**CuI** [89]	-5.84	1.34	3.02	-1.68
**TiO_2@50%CP4**	-5.25	0.75	3.55	-2.80
